# Cuticular waxes of nectarines during fruit development in relation to surface conductance and susceptibility to *Monilinia laxa*

**DOI:** 10.1093/jxb/eraa284

**Published:** 2020-06-18

**Authors:** Leandro Oliveira Lino, Bénédicte Quilot-Turion, Claire Dufour, Marie-Noëlle Corre, René Lessire, Michel Génard, Jean-Luc Poëssel

**Affiliations:** 1 INRAE, GAFL, Montfavet, France; 2 INRAE, PSH, Avignon, France; 3 INRAE, Avignon Université, SQPOV, Avignon, France; 4 CNRS UMR 5200, Bordeaux, France; 5 CONICET-National University of La Plata, Argentina

**Keywords:** Brown rot, cuticular waxes, fruit conductance, fruit defence, fruit development, phenolic derivatives, *Prunus persica*, triterpenoids

## Abstract

The cuticle is composed of cutin and cuticular waxes, and it is the first protective barrier to abiotic and biotic stresses in fruit. In this study, we analysed the composition of and changes in cuticular waxes during fruit development in nectarine (*Prunus persica* L. Batsch) cultivars, in parallel with their conductance and their susceptibility to *Monilinia laxa*. The nectarine waxes were composed of triterpenoids, mostly ursolic and oleanolic acids, phytosterols, and very-long-chain aliphatics. In addition, we detected phenolic compounds that were esterified with sugars or with triterpenoids, which are newly described in cuticular waxes. We quantified 42 compounds and found that they changed markedly during fruit development, with an intense accumulation of triterpenoids during initial fruit growth followed by their decrease at the end of endocarp lignification and a final increase in very-long-chain alkanes and hydroxylated triterpenoids until maturity. The surface conductance and susceptibility to *Monilinia* decreased sharply at the beginning of endocarp lignification, suggesting that triterpenoid deposition could play a major role in regulating fruit permeability and susceptibility to brown rot. Our results provide new insights into the composition of cuticular waxes of nectarines and their changes during fruit development, opening new avenues of research to explore brown rot resistance factors in stone fruit.

## Teaser

Changes in the composition of nectarine cuticular waxes along with variations in fruit conductance and brown rot susceptibility during development suggest a role for cuticular waxes in fruit permeability and defence.

## Introduction

Peaches and nectarines (*Prunus persica* L. Batsch) are among the most highly produced fleshy fruit in the world, and they are important to the agricultural economies of many Mediterranean countries (FAOSTAT, http://www.fao.org/faostat/). These fruits are highly perishable and primarily consumed fresh. They can be stored for only a few days and are susceptible to fungal diseases. Brown rot, which is caused by *Monilinia* spp., is one of the most damaging fungal diseases of stone fruit, both pre- and post-harvest. It can provoke severe yield losses, and few means of control are available other than chemical treatments (Obi *et al.*, 2019). *Monilinia* spp. have often been considered to be opportunistic fungi that can enter the plant tissue via naturally occurring entry points, such as the stomata and skin microcracks (Gibert *et al.*, 2009; Lino *et al.*, 2016), but hyphae could also enter via direct penetration, through degradation of the cuticle by cutinases and of epidermal cell walls by degrading enzymes (Bostock *et al.*, 1999; Lee and Bostock, 2007; Garcia-Benitez *et al.*, 2019). It has long been known that resistance factors to *Monilinia* could be present in the epicarp. Marked differences in infection probability were observed between cultivars of peaches, plums, and apricots following spore deposition on uninjured fruit, whereas the differences were smaller when using artificially injured fruit and were not correlated with the variations observed in intact fruit (Pascal *et al.*, 1994). This finding suggests a primary role for the fruit epicarp in brown rot resistance. In addition, *Monilinia* spores stay quiescent on the surface of immature peaches, which probably produces antifungal compounds that inhibit their development. This lack of fungal development may be due to the suppression of *Monilinia* cutinase expression by caffeic acid derivatives, such as the chlorogenic acid and its isomers that are present in the fruit epidermis and underlying cell layers (Bostock *et al.*, 1999; Lee and Bostock, 2007). However, little is known about the composition of the peach surface itself, which constitutes the outermost layer of the fruit and interacts with *Monilinia* first.

The surface of fruit skin is formed by the cuticle, an extracellular hydrophobic coating composed of a cutin polyester polymer, which primarily consists of esterified fatty acids and cuticular waxes. Cuticular waxes are embedded in the cutin (intracuticular waxes) or they form a crystalline and amorphous coating covering the fruit surface (epicuticular waxes) (Yeats and Rose, 2013). These waxes are complex mixtures of lipids, mostly made up of triterpenoids, phytosterols, flavonoids, and very-long-chain aliphatics, including fatty acids, alkanes, aldehydes, ketones, and alcohols (Yeats and Rose, 2013; Lara *et al.*, 2015). The composition of cuticular waxes shows great variation among and within plant species, during the growth and development stages of the plant organs, and in response to varying environmental conditions.

The cuticle forms the first protective barrier involved in plant defence against biotic and abiotic stresses (Yeats and Rose, 2013). In fleshy fruit, the cuticle is important for reducing fruit transpiration and contributes to post-harvest storability (Lara *et al.*, 2014). The cuticle also plays a major role in plant resistance to many pests and pathogens, not only as a mechanical barrier but also as an active layer that experiences dynamic changes during plant–pathogen interactions. As well as containing antimicrobial compounds, such as terpenoids and flavonoids, the cuticle is also a source of signals perceived by pathogens, activating the production of virulence factors such as cutinases required for infection, and by the host, triggering local and systemic defence responses. There have been significant advances in recent years in understanding the role of the cuticle in plant–pathogen interactions, as summarized in reviews by Reina-Pinto and Yephremov (2009), Łaźniewska *et al*. (2012), Serrano *et al.* (2014), Aragón *et al.* (2017) and Ziv *et al.* (2018).

Cuticle formation in fruit has been reviewed with respect to its composition and organization and the genetic regulation underlying the assembly of its layers throughout fruit development, and these factors have been compared between fruit species (Hen-Avivi *et al.*, 2014; Martin and Rose, 2014; Lara *et al.*, 2015; Trivedi *et al.*, 2019). Nevertheless, with respect to stone fruits, there are only a few published studies about the cuticles of cherries (Peschel *et al.*, 2007), plums (Ismail *et al.*, 1977), and peaches (Belge et al., 2014b, 2019). In peaches, nothing is known about the changes in the cuticle during fruit development. Previous studies that focused on the maturity stage and post-harvest storage were conducted on pubescent fruit only. It was previously shown that the composition of the cuticular layer of trichomes present on pubescent peach skin greatly differs from that of the underlying cuticle (Fernandez *et al*., 2011). Thus, the cuticular wax composition of a glabrous nectarine may differ greatly from that of a pubescent peach.

In this context, we studied the cuticular wax composition of nectarine and its changes during fruit development, in parallel with its conductance and its susceptibility to *Monilinia laxa*, to shed light on the contribution of cuticular waxes to transpiration limitations and to brown rot resistance.

## Materials and methods

### Plant material

Summergrand, a midseason yellow-fleshed cultivar, Zéphir, a midseason white-fleshed cultivar, and Magique, an early white-fleshed cultivar, were grown in an experimental orchard located at the INRAE Research Center in Avignon, France (43°57’00” N, 4°49′01″ E) following standard cultural practices, including pruning and fruit thinning and chemical spray programmes. However, no fungicidal treatments against *Monilinia* spp. were applied after flowering time.

### Fruit sampling

Summergrand and Zéphir fruits were sampled over 2 years during the course of their development, on seven dates in 2012 and five dates in 2015. Magique fruits were harvested only in 2015, on five dates. For each date, samplings of Summergrand and Zéphir cultivars were carried out 1 or 2 days apart except for the last harvest, which corresponded to the date of maturity of each cultivar.

At each date, fruits without visible wounds or rot that were homogeneous in size were harvested. The masses and three dimensions (cheek, suture and height diameters) of the fruit were measured. For biochemical analyses, five lots per date and per cultivar were analysed. The number of fruit per lot depended on their size, ranging from five fruit at the beginning of development to only one fruit at maturity, which represented approximately the same cuticular surface analysed per lot throughout fruit development. For the infection tests, 20 fruits per cultivar were immediately infected in the laboratory at each sampling date during both years. To study the cuticular conductance, 20 fruits per cultivar were harvested over the course of fruit development on seven dates in 2012, four dates in 2013, two dates in 2014, and three dates in 2015 for Summergrand and Zéphir. For Magique, 20 fruits were harvested on eight dates in 2014.

### Cuticular conductance

The fruit conductance to water vapour was estimated during fruit development using total fruit transpiration measurements made over 4 years (2012, 2013, 2014, and 2015). Freshly harvested fruits were measured as described above and placed in a ventilated chamber. The temperature and relative humidity of the chamber were recorded continuously (Log 1520, Sefram, St Etienne, France). Each fruit was weighed every 90 min over approximately 7 h. The method used to calculate the hourly cuticular conductance from the water loss by transpiration is detailed in Gibert *et al.* (2005).

Because the cuticular conductance is very sensitive to the fruit mass, especially when the fruit is small, the mean cuticular conductance of the fruits used for biochemical analyses presented here was predicted from the fruit mass using a smoothed relationship between the mass and the cuticular conductance for each cultivar. Thus, for every lot of harvested fruit, we interpolated the cuticular conductance from the corresponding mean fruit mass. This interpolation was then used to explore the links between the cuticular conductance and the compounds.

### Susceptibility to *Monilinia laxa*

The isolate of *M. laxa* (Ml3) used in this study was obtained from a monospore taken from an apricot fruit mummy on 25 March 2011, which was stored at –20 °C and kept in a glycerol solution in aliquots of 45 μl. The isolate was multiplied by dispensing 5 μl aliquots on to a Petri dish containing modified V8 agar (200 ml V8 juice, 1 g CaCO_3_, 2 g glucose, 2 g yeast extract, 40 g agar, and 1 litre distilled water) and incubated at 25 °C with a 12 h/12 h dark/light cycle for 15 days. Conidial suspensions were prepared before each infection test by washing the colonies with sterile distilled water and one drop of Tween 80 for each Petri dish. The number of spores in a 1/10 dilution of the stock solution was counted on a Malassez cell under the microscope to estimate and adjust the spore concentration of the suspension to 10^5^ conidia ml^−1^. The viability and the probability of germination were verified for each suspension on potato dextrose agar medium.

Before the intentional infection, the fruit were disinfected in a water bath at 55 °C for 45 s and placed inside transparent acrylic plastic boxes in a growth chamber (16 h light at 24 °C and 8 h dark at 18 °C). A 10 µl drop of *M. laxa* suspension at 10^5^ conidia ml^−1^ was deposited on the equatorial zone of a fruit cheek. High humidity was guaranteed in the closed boxes by adding cups of water. The infection status of each fruit (i.e. healthy or infected) was recorded daily over 6 days.

### Extraction of surface compounds

The fruits were washed with reverse-osmosis-treated water and dried. They were then immersed in chloroform (VWR, Normapur) for 30 s under agitation. The extract was filtered through a paper filter and then divided into aliquots for analysis of secondary compounds by high-performance liquid chromatography (HPLC) and analysis of lipids by gas chromatography-mass spectrometry (GC-MS) (in 2012), or used only for HPLC analyses (in 2015).

For HPLC, 10 µg taxifolin (Extrasynthèse Genay, France, prepared in methanol) was added to the aliquot as an internal standard. The extract was concentrated in a rotary evaporator nearly to dryness, and the last millilitre was evaporated under an argon atmosphere to obtain a dry residue, which was solubilized in 1 ml methanol (VWR Hypersolv Chromanorm) and then filtered on a 0.45 µm polytetrafluoroethylene membrane and stored at 4 °C until analysis. Special attention was paid to the verification of any precipitation in the vial.

For the GC-MS analysis, 10 µg docosane (Sigma-Aldrich, Saint-Quentin Fallavier, France, prepared in chloroform) was added to the aliquot as an internal standard, and the same procedure as described above was followed for concentration. The dry residue was weighed and stored at 4 °C until analysis.

### HPLC-mass spectrometry identification

The phenolic compounds and triterpenoids were identified by HPLC coupled with electrospray ionization-mass spectrometry (HPLC/ESI-MS) using a Waters ACQUITY UPLC system (Waters, Milford, MA, USA) coupled to an UV/Vis diode-array detector and an HCT ultra ion trap mass spectrometer equipped with an electrospray ionization source (Bruker Daltonics, Bremen, Germany). The HPLC conditions were the same as those used for the quantitative analyses (see below). Mass detection was conducted in negative electrospray ionization mode using *m/z* values from 100 to 1000. The MS conditions were as follows: capillary voltage 2 kV, nitrogen flow rate 10 litres min^−1^, desolvation temperature 365 °C, and nebulization pressure 50 psi.

The compounds were characterized according to their UV and mass spectra and their retention times. Co-chromatography was performed with known standards when available (ursolic and oleanolic acids, Extrasynthèse, Genay, France; corosolic and maslinic acids, Sigma-Aldrich, Saint-Quentin Fallavier, France).

### Quantitative HPLC analyses

Quantitative HPLC analyses were performed with a Shimadzu Prominence HPLC system equipped with a reversed-phase C18 column (Merck LiChroCART^®^ 250 mm × 4 mm packed with Superspher RP-18 endcapped, 4 µm) coupled to a photodiode array detector. The mobile phase was a mixture of solvent A [ultrapure water (Millipore Synergy-UV) acidified to pH 2.6 with orthophosphoric acid at 85% (VWR Normapur) and filtered with a Millipore Durapore HVLP04700 0.45 µm membrane] and solvent B [methanol (VWR Hypersolv Chromanorm)].

To separate all the free terpenoids and their derivatives, elution was performed with a linear gradient from 35% to 80% B in 28 min, followed by a 15 min isocratic elution with 80% B, a linear gradient to 90% B in 5 min, a 10 min isocratic elution with 90% B, a linear gradient to 100% B in 5 min, and a 5 min isocratic elution with 100% B. The column temperature was set to 30 °C and the flow rate was 0.7 ml min^−1^. The chromatograms were analysed at 210 nm for free triterpenoids and 315 nm for phenolic derivatives.

For the quantitative analysis, a calibration curve was obtained by injecting known concentrations of standard compounds. Unidentified triterpenoids were expressed as ursolic acid. For the *p*-coumaroyl derivatives, *p*-coumaric acid was used as the standard (Sigma-Aldrich, Saint-Quentin Fallavier, France). The results were expressed in µg per unit of fruit surface and per fruit.

### GC-MS analyses

The extracts were derivatized and analysed by GC-MS as described by Bourdenx *et al.* (2011). The samples were analysed with a Hewlett-Packard 5890 series II equipped with a flame ionization detector and a 30 m × 0.32 mm HP-1 capillary column with helium as the carrier gas. Quantification was performed by taking the ratios of the flame ionization detector peak areas to the area of the internal standard. The results were expressed in µg per unit of fruit surface area and per fruit.

### Statistical analysis

The data were analysed using R software v.3.1.2 (R Core Team, 2014). The ‘loess’ function, which allows for adjustment by local polynomial regression, was used to add trends showing the relationships between two variables on the graphs and to predict the cuticular conductance from the fruit mass.

To detect significant differences between the cultivars and the effect of time on growth and on the course profile of the compounds, a generalized linear mixed-effects model (GLMM) was applied as described in Bugaud *et al.* (2012). The lmer function in the ‘lmer4’ library was used. Details of the statistical results are given in Supplementary Datasets S1 and S2 at *JXB* online. A complete model including the effect of days after bloom (DAB) and the effect of cultivar as well as the quadratic and cubic terms of the DAB was compared with the simplest models. The effect of year was tested on a subset of data from which the data for the Magique cultivar had been removed. For surface compounds, the effect of the years was added to the complete model and tested. A comparison of the nested models was performed with a likelihood ratio test using the ANOVA function in R. The threshold of significance was set at 0.01. Predictions from GLMMs with significant effects only were added to data plots. Polynomial effect in GLMM analysis was used to determine the shape of the relationships between surface conductance or infection probability and compounds. In case quadratic and cubic terms were significant, non-linear correlations were estimated using the nlcor function in R (GitHub ‘ProcessMiner’). This returned an absolute non-linear correlation estimate and corresponding adjusted *P*-value, and the relationships were added on the plots (see Supplementary Datasets S1 and S2).

## Results

### Changes in fruit characteristics during development

#### Fruit growth

**Fig. 1.**
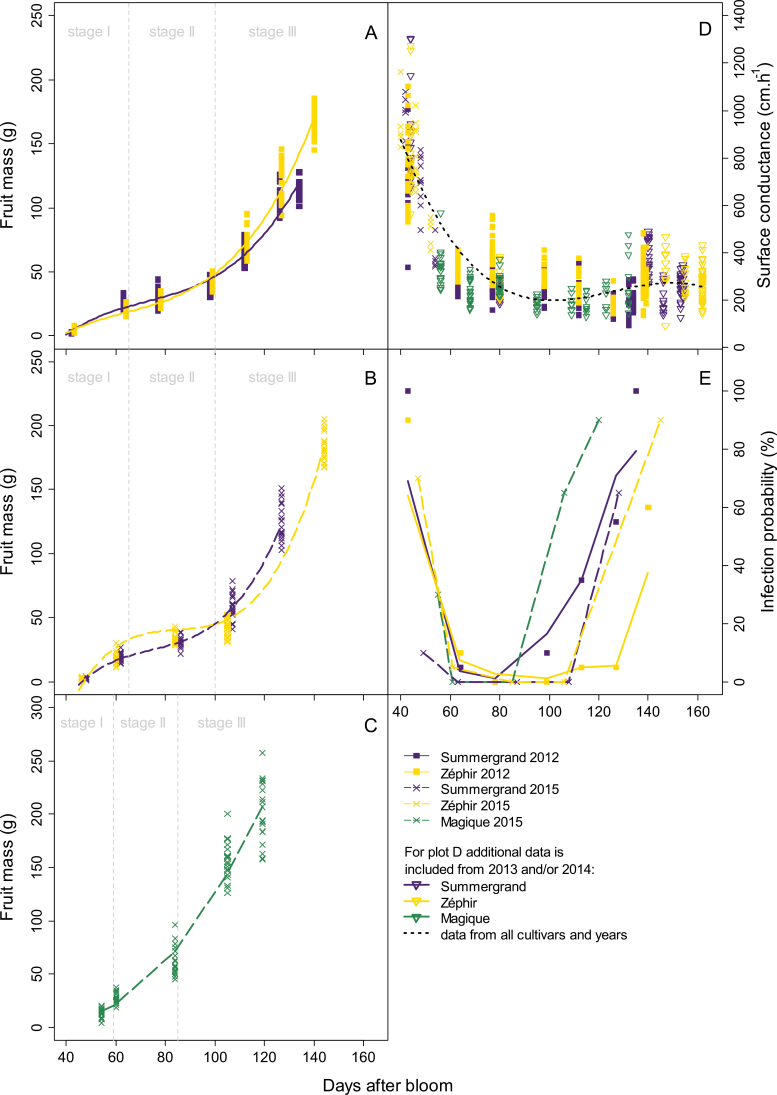
Changes in the fruit fresh mass (A–C), surface conductance (D), and infection probability (E) during nectarine fruit development. DAB, Days after bloom. Filled squares, crosses, and empty triangles correspond to data obtained in 2012, 2015, and both 2013 and 2014, respectively, for three cultivars, Summergrand (indigo), Zéphir (gold), and Magique (green). Solid, dashed, and dotted lines in (A–D) are fitted linear models by GLMM of, respectively, data from 2012 and 2015 for each cultivar (cultivar and year effect interactions are significant) and all data together (no significant cultivar effect or DAB:cultivar interaction; black dotted line in D). Solid and dashed lines in (E) indicate data from 2012 and 2015, respectively, for each cultivar.

The fruits from the three cultivars followed a typical growth curve for stone fruits, with three stages corresponding to (i) the first exponential growth, characterized by a rapid increase in cell division and elongation (stage I); (ii) pit hardening, when the endocarp hardens to form the stone (stage II); and (iii) the second exponential growth, characterized by a rapid increase in fruit size (stage III). The three cultivars had different growth durations and final fruit sizes (significant DAB:cultivar interactions; *P*<2.2e-16) (Fig. 1A–C). In 2015, Magique reached maturity at 119 DAB, whereas Summergrand and Zéphir reached maturity at 127 and 144 DAB, respectively. Despite their shorter duration of growth, the Magique fruits reached similar masses to the Zéphir fruits, of approximately 150–200 g. The Summergrand fruits were smaller, at approximately 100–150 g. There was no simple effect of year on growth between 2012 and 2015 for Summergrand and Zéphir, but there were significant year:cultivar and year:DAB interactions, probably mainly due to the duration of growth (*P*=1.299e-07 and 0.00012, respectively).

#### Fruit cuticular conductance

The permeability of the fruit to water was examined by surveying the total fruit water loss. Temporal curves of fruit surface conductance and fruit growth were constructed by grouping data from the four different years for Summergrand and Zéphir (Fig. 1D, Supplementary Fig. S1). The surface conductance of Summergrand and Zéphir fruits followed a very similar pattern of change during growth, characterized by a strong drop in conductance during stage I [no significant cultivar effect (*P*=0.38) or DAB:cultivar interaction (*P*=0.38), but significant DAB and DAB polynomial terms effects (*P*=2.2e-16)] (Fig. 1D). This feature was particularly visible when plotting the surface conductance against the fruit mass (Supplementary Fig. S1A). Although there is a lack of data for very small fruits, the trend for Magique was very similar to those of the other two cultivars. The surface conductance of very small fruit (smaller than 14 or 15 g) was very high, and it declined sharply with a slight increase in the fruit mass (Supplementary Fig. S1C). The surface conductance remained low during subsequent fruit growth and increased only slightly at the approach towards maturity for all three cultivars (Fig. 1D).

#### Fruit susceptibility to Monilinia laxa

Susceptibility to *M. laxa* during fruit development, as assessed under laboratory conditions, showed a very pronounced variation marked by three successive phases (Fig. 1E) roughly corresponding to the three stages of fruit development. Small fruit exhibited medium to high susceptibility during a short first phase that ended at approximately 62 DAB with fruit reaching approximately 20 g (Supplementary Fig. S1B). This early susceptible phase corresponded well with stage I of fruit development and with the high surface conductance of the young fruit described above (Supplementary Fig. S1C). It was followed by a phase of high resistance, with the infection probability being null for the three cultivars. This phase occurred during endocarp lignification, at a time when the increase in mass was slow (stage II, approximately 63 to 90–108 DAB depending on the cultivar and year). The last phase was characterized by a continuous increase in infection probability during the final fruit growth and ripening period (stage III), ending with very high values at maturity. The start of this increase was dependent on the cultivar and year, whereas the rate of the increase appeared to be quite constant.

### Identification of and changes in surface waxes during fruit development

The dramatic changes in water conductance and susceptibility to *M. laxa* that occurred during fruit development led us to investigate the changes in fruit surface composition that were potentially linked. The surface waxes obtained by a short period of soaking in chloroform included secondary compounds, namely, triterpenoids and phenolic derivatives, and lipids. They were characterized and analysed in cultivars Summergrand and Zéphir throughout fruit development in 2012 by HPLC and GC-MS. HPLC analyses of secondary compounds were repeated in 2015 with the addition of a third cultivar, Magique.

#### Triterpenoids and phenolics

**Fig. 2.**
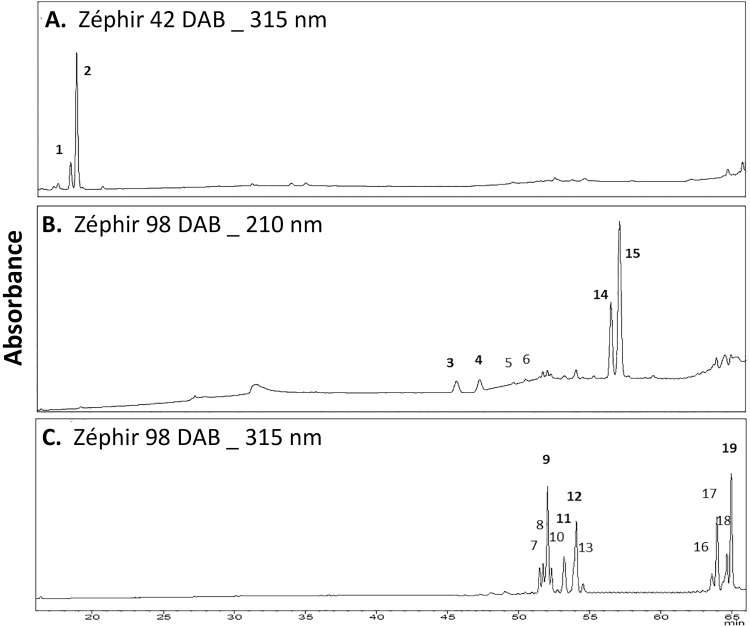
HPLC chromatograms of secondary compounds from cuticular waxes of Zéphir fruit. (A) Sugar esters of *p*-coumaric acid of Zéphir at 315 nm, measured at 42 days after bloom (DAB). (B) Free triterpenoids at 210 nm, measured at 98 DAB. (C) Triterpenoid esters of *p*-coumaric acid at 315 nm, measured at 98 DAB. Peaks correspond to: **1**, **cpdh1**; **2**, **cpdh2**; **3**, **thu1**; **4**, **thu2**; 5, maslinic acid; 6, corosolic acid; 7, cdhu1; 8, cdhu2; **9**, **cdhu3**; 10, cdhu4; **11**, **cdhu5**; **12**, **cdhu6**; 13, fdhu; **14**, **oleanolic acid**; **15**, **ursolic acid**; 16, cou1; 17, cou2; 18, cou3; **19**, **cou4**. Quantified compounds are in bold text. Abbreviations of compound names are defined in Table 1.

Nineteen compounds were detected by HPLC on the fruit surface, and they were present in the three cultivars (Fig. 2). Identification of these compounds by mass spectrometry (Table 1) established the major presence of free triterpenoids. Mass spectrometry also revealed the presence of two families of hydroxycinnamic acid derivatives, which were esterified with either sugars or triterpenoids, showing the opposite pattern of change during fruit development.

**Table 1.** Characteristics of secondary compounds identified by HPLC-DAD-ESI-MS in nectarine fruit surface waxes

Peak	Proposed structure	Abbreviation	RT	λ _ max_	MS (*m/z*)	MS^2^ (*m/z*)	MS^3^ (*m/z*)
**1**	*p*-coumaroylpentacetyldihexoside	cpdh1	18.5	311	697	655, **637**, 613, 595, 391	[637]: **595**, 577, 553, 535, 331, 287
**2**	*p*-coumaroylpentacetyldihexoside	cpdh2	19.0	315	697	655, **637**, 613, 595, 391	[637]: **595**, 577, 553, 535, 331, 287
**3**	trihydroxy-urs-12-en-28-oic acid^*a*^	thu1	45.5	198	487	**409**	[409]: 391, **379**
**4**	trihydroxy-urs-12-en-28-oic acid^*a*^	thu2	47.0	198	487	**469**	[469]: 451, 439, **423**, 387
5	2α,3β-dihydroxyolean-12-en-28-oic acid (maslinic acid) (S)	Mas	49.5	198	471	425, **407**	[407]: **389**, 377, 351, 279, 253, 205
6	2α,3β-dihydroxyurs-12-en-28-oic acid (corosolic acid) (S)	Cor	50.5	198	471	407	[407]: 389, 377, **351**, 279, 253, 205
7	*p*-coumaroyl-2,3-dihydroxy-urs-12-en-28-oic acid^a^	cdhu1	51.5	198/311	617	**573,** 497	[573]**: 529**
8	*p*-coumaroyl-2,3-dihydroxy-urs-12-en-28-oic acid^a^	cdhu2	51.7	198/308	617	**573,** 497	[573]**: 529**
**9**	*p*-coumaroyl-2,3-dihydroxy-urs-12-en-28-oic acid^a^	cdhu3	52.0	198/312	617	573, **497**	[497]: 453, **407**, 391
10	*p*-coumaroyl-2,3-dihydroxy-urs-12-en-28-oic acid^a^	cdhu4	52.3	198/308	617	**573,** 497	[573]**: 529**
**11**	*p*-coumaroyl-2,3-dihydroxy-urs-12-en-28-oic acid^a^	cdhu5	53.0	198/310	617	**573**, 497	[573]: **529**
**12**	*p*-coumaroyl-2,3-dihydroxy-urs-12-en-28-oic acid^a^	cdhu6	54.0	198/308	617	573, **497**	[497]: 453, **407**, 391
13	feruloyl-2,3-dihydroxy-urs-12-en-28-oic acid^a^	fdhu	54.5	198/322	647	632-617-603-588-**497**-453-193	[497]: **453**, 407, 391
**14**	3*β*-hydroxy-olean-12-en-28-oic acid (oleanolic acid) (S)	Ole	56.5	198	455	**407**	[407]: 439, 421, 407, 391, **377**
**15**	3*β*-hydroxy-urs-12-en-28-oic acid (ursolic acid) (S)	Urs	57.0	198	455	**407**	[407]: 439, 421, 407, 391, **377**
16	3β-*p*-coumaroyloxy-urs-12-en-28-oic acid^a^	cou1	63.8	198/311	601	555, **437**	[555]: 525, **367**, 351
17	3β-*p*-coumaroyloxy-urs-12-en-28-oic acid^a^	cou2	64.0	198/308	601	555, **437**	[437]: 423 (tr)
18	3β-*p*-coumaroyloxy-urs-12-en-28-oic acid^a^	cou3	65.0	198/309	601	555, **437**	[437]: 391 (tr) [555]: 539, 525, **351**
**19**	3β-*p*-coumaroyloxy-urs-12-en-28-oic acid^a^	cou4	65.2	198/312	601	555, **437**	[437]: 391, **367**

^*a*^ Ursolic acid or oleanolic acid as principal structure.

Peaks are numbered as in Fig. 2; quantified compounds are in bold. (S), Structure confirmed by standard; RT, retention time in min; λ _ max_, wavelength of absorption maxima. MS gives the parent ion for [M-H]^–^ (*m/z*), MS^2^ and MS^3^ the fragmentations of the parent ion and the given selected ion, respectively. The major fragment ion is in bold. tr, Traces.

#### Free triterpenoids

Free triterpenoids were barely detected on the surface of young fruit (stage I, 41–42 DAB). During the later stages of fruit development, six compounds were identified as oleanolic (peak 14 in Fig. 2 and Table 1) and ursolic (15) acids, the two major compounds, together with their respective 3β-hydroxylated derivatives, tentatively identified as maslinic (5) and corosolic (6) acids by co-chromatography with standards, and two dihydroxylated derivatives of oleanolic or ursolic acids (3 and 4) having a parent ion at *m/z* 487 (Table 1). The nature of the triterpenoid structure of these last two compounds, which are abbreviated thu1 and thu2 in Table 1, was not further determined.

**Fig. 3.**
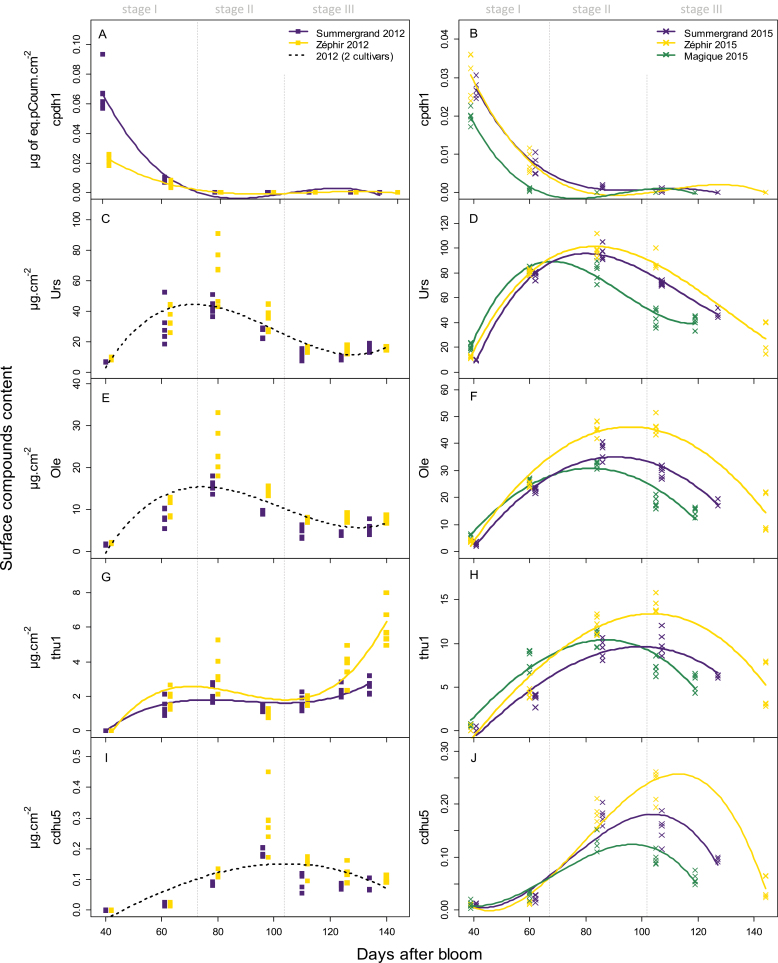
Changes in the content of secondary compounds in nectarine cuticular waxes during fruit development analysed by HPLC. Squares and crosses correspond to data from 2012 and 2015, respectively, for three cultivars: Summergrand (indigo), Zéphir (gold), and Magique (green). Compounds belong to three families: *p*-coumaroyl-acetylsugars (A, B), free triterpenoids (C–H), and *p*-coumaroyl-triterpenoids (I, J). Lines are fitted linear models by GLMM according to significant cultivar and DAB (quadratic and cubic terms) effects. The fruit growth stages marked are those of Summergrand and Zéphir cultivars. See Table 1 for definitions of abbreviations for compound names, Supplementary Fig. S2 for changes in other quantified secondary compounds during fruit development, and Supplementary Dataset S1 for GLMM results.

All the quantified free triterpenoids were highly accumulated before the second sampling date, at approximately 60 DAB (i.e. during stage I), and they continued to accumulate at the beginning of stage II (Fig. 3, Supplementary Figs S2 and S4, Supplementary Table S1). These compounds reduced in content later, at the end of stage II. In 2012, they tended to increase again during maturation, especially thu1 and thu2, which overall represented 20.6% and 34.2% of the free triterpenoids at maturity for Summergrand and Zéphir, respectively. This pattern of change was similar in 2012 and 2015, although in 2012, the amounts were slightly lower. The same pattern was observed for the three cultivars, with Magique showing faster accumulation, especially for thu1 and thu2, most likely because of the shorter development time of the fruit of this cultivar.

#### p-Coumaroyl-acetylsugars

The first family of hydroxycinnamic derivatives consisted of two isomers of *p*-coumaric acid derivatives esterified with a dihexose moiety, which were identified by ESI-MS as *p*-coumaroylpentacetyldihexosides (1, cpdh1 and 2, cpdh2; Fig. 2, Table 1). Their structural assignments were based on the double loss in fragmentation of 42 amu that is typical of the acetyl group, a fragment ion at *m/z* 535 in MS3 resulting from the loss of the *p*-coumaroyl unit, and their λ _max_ at 311 nm and 315 nm, respectively. The identical fragmentation pattern and their slight difference in λ _ max_ could indicate *cis* and *trans* stereoisomers of the same compound. Strikingly, cpdh1 and cpdh2 were almost exclusively present at the earliest stage of fruit development, when they were the primary phenolics detected (Fig. 2), and they disappeared at later stages (Fig. 3, Supplementary Figs S2 and S4, Supplementary Table S1).

#### Hydroxycinnamoyl-triterpenoids

In the second family, 11 hydroxycinnamic esters of triterpenoids were detected at both 210 nm and 315 nm, forming two clusters according to their retention time (Fig. 2). Among the seven compounds in the first group, six were *p*-coumaroyl derivatives of hydroxy-oleanolic or -ursolic acids (peaks 7–12; Fig. 2, Table 1). They displayed a parent ion at *m/z* 617 and a primary fragment ion at *m/z* 573 (loss of CO_2_) or *m/z* 497 (loss of HOPhCH=CH) in MS^2^. The latter fragment arises from the formation of a carbonate intermediate and thus highly suggests a 2,3-dihydroxylation scheme, as in maslinic or corosolic acids. The two most abundant derivatives (9 and 12) even displayed a fragment ion at *m/z* 453 (loss of *p*-coumaric acid) in MS^3^. The nature of the triterpenoid structure and the position of the acyl group were not further determined. These compounds were named *p*-coumaroyl-2,3-dihydroxy-urs-12-en-28-oic acids and were numbered cdhu1 to cdhu6 according to their retention time (Fig. 2, Table 1). The different derivatives detected may correspond to *cis* and *trans* stereoisomers like those identified in apple peels (He and Liu, 2007; McGhie *et al.*, 2012). This hypothesis is supported by the slight difference in the λ _max_ of the compounds, and 308 nm could be indicative of *cis* isomers whereas 310/311 nm could correspond to *trans* isomers (Mitani *et al.*, 2018). The last compound of the group (13), showing a λ _max_ at 322 nm, appeared to be a feruloyl derivative of hydroxy-ursolic or -oleanolic acid. Its parent ion at *m/z* 647 fragmented to yield a major fragment at *m/z* 497 in MS^2^ (loss of HO(MeO)PhCH=CH), which itself gave a fragment at *m/z* 453 (loss of CO_2_) in MS^3^. It was named feruloyl-2,3-dihydroxy-urs-12-en-28-oic acid (fdhu; Table 1).

The four compounds in the second group (peaks 16–19) were *p*-coumaroyl derivatives of ursolic or oleanolic acids. They were identified based on their parent ion at *m/z* 601 and a major fragment ion at *m/z* 437 (loss of *p*-coumaric acid) in MS^2^. They were named 3β-*p*-coumaroyloxy-urs(olean)-12-en-28-oic acids and numbered cou1 to cou4 according to their retention time (Fig. 2, Table 1). As hypothesized for the first group, the different derivatives may correspond to *cis* and *trans* stereoisomers.

Quantification was performed for the major *p*-coumaroyl derivatives of triterpenoids, namely, cdhu3, cdhu5 and cdhu6 in the first group and cou4 in the second group (Fig. 3, Supplementary Figs S2 and S4, Supplementary Table S1). All these compounds were almost absent at the beginning of fruit development and accumulated on the fruit surface during endocarp lignification (79–80 DAB), later than the free triterpenoids. They culminated at the end of stage II or the beginning of stage III (97–98 and 105–107 DAB) and then decreased sharply until maturity. Unlike the free triterpenoids, the contents of their phenolic derivatives were roughly similar in 2012 and 2015. There were no significant differences between the cultivars except for Magique fruit, which showed an earlier decrease in these compounds, most likely due to its faster development, and a lower amount of cou4 than the other cultivars.

#### Lipids and other compounds

**Fig. 4.**
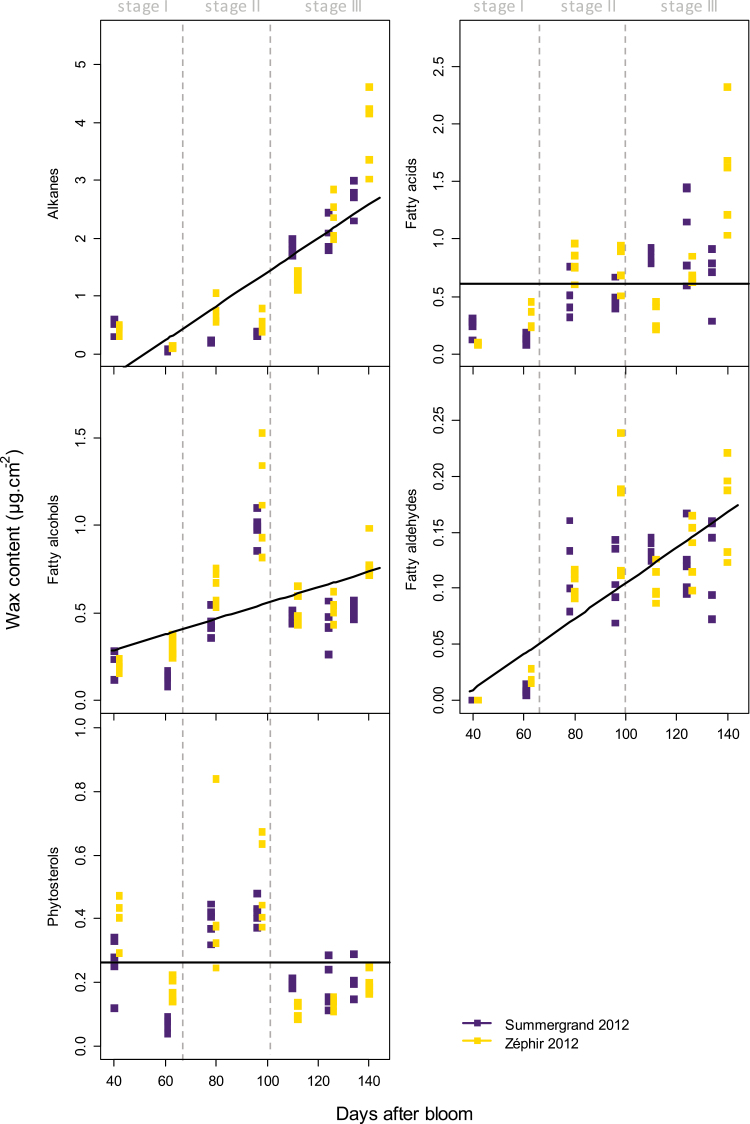
Changes in the content of different classes of lipids in nectarine fruit cuticular waxes during fruit development, analysed by GC-MS in 2012, from the cultivars Summergrand (indigo) and Zéphir (gold). Lines are fitted linear models by GLMM according to significant DAB effect (no significant cultivar effect or quadratic or cubic DAB effects). See Supplementary Dataset S1 for GLMM results.

Thirty-two lipid compounds were detected by GC-MS from the surfaces of Summergrand and Zéphir fruit in 2012 and were grouped into five classes: alkanes, fatty acids, fatty alcohols, fatty aldehydes, and phytosterols (Fig. 4, Supplementary Table S1, Supplementary Fig. S4). The major presence of triterpenoids previously identified by HPLC was confirmed by GC-MS. However, they are not considered here since they were already well determined by HPLC analyses.

The dynamics of the different classes of lipids were very similar for both cultivars (Fig. 4) and they showed a remarkable pattern of change during fruit development. In fact, the DAB effect was significant for three classes, the alkanes, aldehydes, and alcohols; the fatty acids and phytosterols can be considered to be constant over time. No significant effect of cultivar or interaction between cultivar and time was detected.

Alkanes were the primary class of lipids at maturity (Fig. 4), with a predominance of very-long-chain compounds with an odd number of carbons (C23 to C29) (Supplementary Table S1, Supplementary Fig. S4). Unlike the triterpenoids, the amounts of these molecules were low until the end of endocarp lignification. They began to accumulate only at 111–113 DAB and during final fruit growth, to reach concentrations at maturity of approximately 2.7 µg cm^−2^ for Summergrand and 3.9 µg cm^−2^ for Zéphir (Fig. 4, Supplementary Table S1).

The contents of fatty acids were very low at 41–42 DAB. A non-significant tendency for them to increase until ripening was observed (Fig. 4). For both cultivars, the proportion of the different fatty acids changed during fruit development: while C16 and C18 fatty acids predominated in young fruit, longer chains appeared later, with the C22, C24, and C26 compounds being the most prominent at maturity (Supplementary Table S1, Supplementary Fig. S4).

The fatty alcohols were low in content at the beginning of fruit development (41–42 DAB) (Fig. 4). Very-long-chain compounds (C24 to C30) increased in both cultivars from 62–63 to 97–98 DAB (Supplementary Table S1, Supplementary Fig. S4). They then tended to decrease until maturity. *n*-Hexacosanol (C26) and *n*-octacosanol (C28) were the primary compounds at maturity.

Fatty aldehydes were also represented by very-long-chain compounds (C18, C22, C24, and C30), with the C30 compound triacontanal being the most important (Supplementary Table S1, Supplementary Fig. S4). They were almost absent at the first two sampling dates and accumulated slightly later (Fig. 4).

The phytosterols were primarily represented by stigmasta-3,5-diene at the beginning of fruit development (Fig. 4, Supplementary Table S1, Supplementary Fig. S4). While the content of this compound sharply decreased at the later dates, the amount of β-sitosterol increased, following the dynamics of the triterpenoids and constituting the major phytosterol at maturity.

### Global variations in nectarine waxes during fruit development

**Fig. 5.**
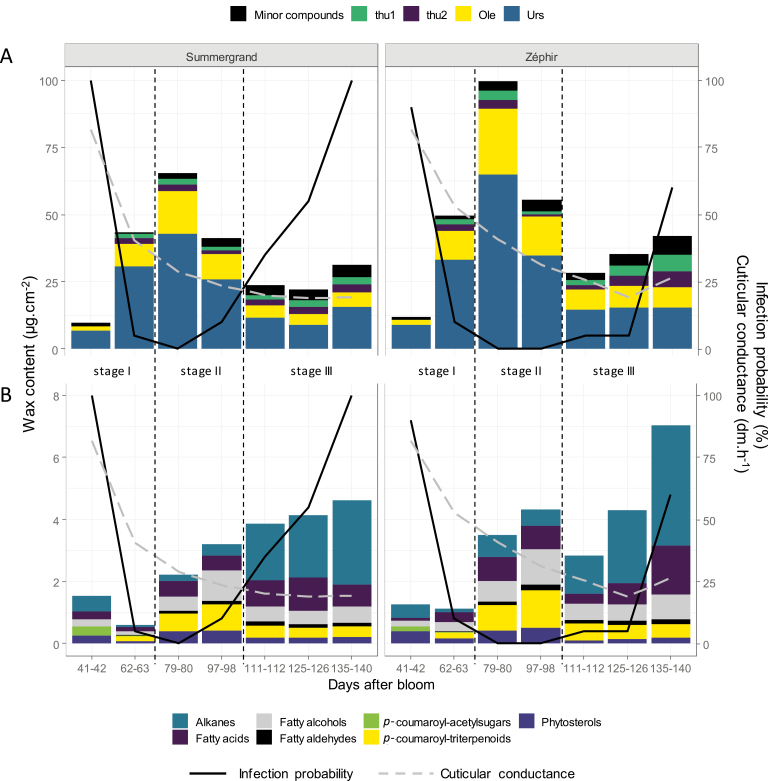
Changes in the classes of cuticular wax compounds from two nectarine cultivars, Summergrand (left) and Zéphir (right), analysed in 2012 during fruit development (measured in days after bloom). (A) Main classes of wax compounds. ‘Minor compounds’ is the sum of all compounds except free terpenoids (thu1, thu2, Ole, Urs). (B) ‘Minor compounds’ only (*p*-coumaroyl-triterpenoids, *p*-coumaroyl-acetylsugars, alkanes, fatty acids, fatty alcohols, fatty aldehydes, and phytosterols). Surface conductance (grey dotted line) and infection probability (black line) are also indicated.

Considering the sum of the compounds extracted from the surface of nectarine fruit in 2012, total waxes were very low at the beginning of fruit development, at 9.9 and 12.1 µg cm^−2^ for Summergrand and Zéphir, respectively, at 41–42 DAB (Fig. 5, Supplementary Table S1). Subsequently, the waxes accumulated strongly at the beginning of endocarp lignification to reach a 6- to 8-fold increase by the middle of stage II (65.5 and 99.7 µg cm^−2^ at 79–80 DAB). They then decreased until the end of stage II (23.8 and 28.5 µg cm^−2^ at 111–112 DAB) and increased slightly again as ripening approached (31.2 and 42.1 µg cm^−2^ at maturity, 135–140 DAB). A polynomial trend of the shape was significant compared with the linear trend (*P*<2.2e-16). The total wax content per fruit showed a similar pattern of change during fruit development (Supplementary Fig. S3). The kinetics and amounts of total waxes were very similar between the two cultivars, and no significant effect of cultivar (*P*=0.97) or interaction between cultivar and time (*P*=0.46) was detected.

Throughout fruit development, the free triterpenoids were the predominant class of surface compounds for both cultivars Summergrand and Zéphir, constituting 81.4–98.6% of the total surface compounds depending on the developmental stage and the cultivar (Table 2). The levels of free triterpenoids were very low at the beginning of fruit development and highest at approximately 80 DAB, in the middle of stage II (Fig. 5A). These compounds primarily contributed to the typical bell curve kinetics of surface waxes. Ursolic acid was always the primary compound, but its relative proportion diminished throughout fruit development.

**Table 2.** Changes in the proportion (%) of each class of wax compounds from the surface of Summergrand and Zéphir nectarines during fruit development in 2012

	Summergrand							Zéphir						
**DAB**	**41**	**62**	**79**	**97**	**111**	**125**	**135**	**42**	**63**	**80**	**98**	**112**	**126**	**140**
Free triterpenoids	84.56	98.61	96.58	92.27	83.74	81.43	85.28	89.41	97.74	96.48	92.21	89.97	87.75	83.30
*p*-coumaroyl-acetylsugars	2.92	0.07	0.00	0.00	0.00	0.00	0.00	1.24	0.04	0.00	0.00	0.00	0.00	0.00
*p*-coumaroyl-triterpenoids	0.00	0.39	0.87	2.04	1.64	1.44	1.09	0.00	0.36	0.82	2.20	1.93	1.33	1.02
Alkanes	4.94	0.16	0.34	0.85	7.66	9.00	8.68	3.56	0.24	0.71	0.97	4.39	6.67	9.22
Fatty acids	2.52	0.30	0.76	1.19	3.58	4.86	2.24	0.75	0.63	0.79	1.33	1.09	1.96	3.75
Fatty alcohols	2.32	0.30	0.67	2.40	1.98	1.93	1.67	1.66	0.61	0.65	2.07	1.86	1.50	1.88
Fatty aldehydes	0.00	0.02	0.17	0.27	0.55	0.54	0.42	0.00	0.04	0.11	0.31	0.35	0.37	0.40
Phytosterols	2.72	0.16	0.60	1.02	0.84	0.85	0.67	3.31	0.36	0.43	0.92	0.39	0.40	0.45

DAB, Days after bloom.

The other compounds, namely, *p*-coumaroyl-acetylsugars, *p*-coumaroyl-triterpenoids, and lipids, accounted for a small portion of the total waxes. In contrast to the free triterpenoids, their relative proportion was highest at the beginning of fruit development and during stage III (Table 2), with maximum content reached in stage III (Fig. 5B). In addition to free triterpenoids, alkanes were always the primary compounds during these two periods, representing 3.6–4.9% of the total in young fruit and 8.7–9.2% at maturity depending on the cultivar (Table 2).

### Relationships between surface waxes and fruit characteristics

#### Changes in cuticular conductance during fruit development

**Fig. 6.**
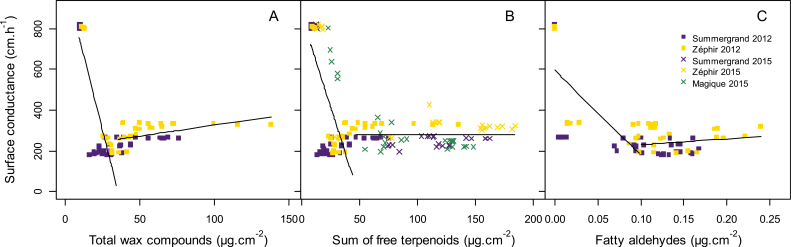
Relationships between surface conductance (calculated from the corresponding fruit mass) and cuticular wax compounds. (A) Total wax compounds, (B) sum of free terpenoids (Ole, Urs, thu1, and thu2), and (C) total fatty aldehydes. Squares and crosses indicate data from 2012 and 2015, respectively, for three nectarine cultivars, Summergrand (indigo), Zéphir (gold), and Magique (green). Lines indicate relationships obtained from non-linear correlation analysis. See Supplementary Dataset S2 for detailed statistical results.

The surface conductance displayed a strong decrease during stage I. Looking at the total wax compounds or, more specifically, the sum of free triterpenoids or other compounds such as fatty aldehydes, their trends during the beginning of fruit development suggest potential explanations for the marked decrease in the surface conductance (Fig. 6). In fact, the cuticular conductance displayed high values for low amounts of these compounds. When these compounds exceeded a threshold value, the cuticular conductance decreased and then remained low. The GLMM analysis of polynomial effects showed significant effects of quadratic and cubic terms of the compounds on surface conductance. The non-linear trends were confirmed by significant non-linear correlations (Supplementary Dataset S2). The non-linear correlation (|*r*|=0.32) between the sum of free terpenoids and surface conductance was highly significant (adjusted *P* =8.09e-10).

#### Susceptibility to Monilinia laxa

**Fig. 7.**
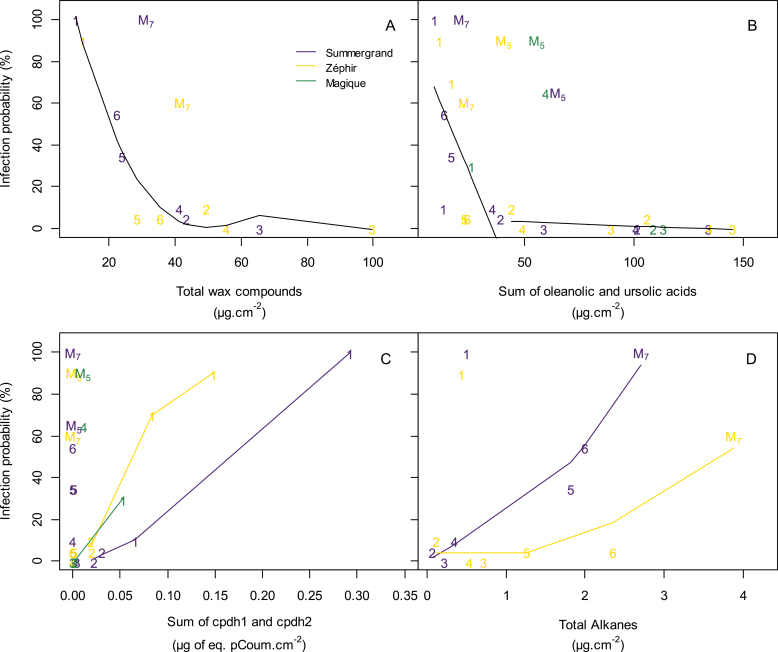
Relationships between probability of infection with *Monilinia laxa* and cuticular wax compounds from the nectarine cultivars Summergrand (indigo), Zéphir (gold), and Magique (green). (A) Total wax compounds, (B) sum of oleanolic and ursolic acids, (C) total *p*-coumaroyl-acetylsugars at immature stages, and (D) total alkanes at maturity stages. Numbers refer to observed data for different harvests; M refers to the last harvest at maturity (M_7_, harvest 7 in 2012; M_5_, harvest 5 in 2015). In (A), the line is the fitted linear model by GLMM on data from 2012 from all harvests except M; in (B), lines are relationships obtained from non-linear correlation analysis on data from all harvests except M for Summergrand and Zéphir, and all harvests except 4 and M for Magique; in (C), lines were obtained by smoothing together data from 2012 and 2015 for harvests 1 and 2 for each cultivar; in (D), lines were obtained by smoothing data from 2012 for all harvests except 1 for each cultivar. See Supplementary Dataset S2 for detailed statistical results.

As shown above and in Fig. 5, the infection probability changed markedly during fruit development in the same way as the other fruit characteristics studied in this work. We therefore analysed the potential relationships between infection probability and the quantity and composition of cuticular waxes (Fig. 7), knowing that factors influencing susceptibility to *Monilinia* may differ during fruit development. A clear negative relationship between the total cuticular waxes measured in 2012 and brown rot susceptibility was observed for the cultivars Summergrand and Zéphir (Fig. 7A), with the exception of the ripe fruit sampled at maturity (harvest M), which was excluded from the curve. The same negative relationship was observed when considering the contents of ursolic and oleanolic acids analysed in 2012 (cultivars Summergrand and Zéphir) and in 2015 (cultivars Summergrand, Zéphir, and Magique) (Fig. 7B), again except for the maturity stage, primarily in 2015, and additionally for pre-maturity harvest 4 of Magique, which has particularly large fruit and early ripening. Again, the GLMM analysis of polynomial effects showed significant effects of quadratic and cubic terms of the total cuticular waxes on the infection probability (*P*=7.789e-05 and 0.000482, respectively). Unfortunately, owing to the lack of available data when considering integrative variables as probability (no repetition was possible at each sampling date), the non-linear correlation analysis could not be applied. Considering the relationship between the sum of the contents of oleanolic and ursolic acids and infection probability (*P*=0.001 and 0.005, respectively), more data were available (2 years, 3 cultivars), allowing a non-linear correlation analysis. In this case, a non-linear correlation (|*r*|=0.35; *P*=0.015) was confirmed (Fig. 7B, Supplementary Dataset S2).

By contrast, we found a positive relationship between the infection probability and *p*-coumaroyl-acetyl sugars (cpdh1 and 2) for all three cultivars (Fig. 7C) when these compounds were present at the beginning of fruit development (harvests 1 and 2) (Summergrand: *r*=0.99, *P*=0.003; Zéphir: *r*=0.97, *P*=0.03; Magique: insufficient data). A positive relationship was similarly observed between the alkane contents and susceptibility to brown rot, especially when the level of alkanes increased during the final stage of fruit development (Fig. 7D) (Summergrand: *r*=0.95, *P*=0.004; Zéphir: *r*=0.8, *P*=0.04) except for harvest 1, in which the alkane contents were low and other susceptibility factors may have intervened.

## Discussion

This work investigated cuticular waxes from the fruit surfaces of three nectarine cultivars during fruit development in relation to changes in their cuticular conductance and susceptibility to *Monilinia*.

### Predominant free triterpenoids and new phenolic derivatives in nectarine cuticular waxes

To our knowledge, this study is the first to reveal the cuticular wax composition of nectarine fruit. At maturity, the total wax amounts ranged from 31 to 42 µg cm^−2^ depending on the cultivar (Supplementary Table S1), much lower than the values obtained for peaches by Belge *et al.* (2014b) after an exhaustive extraction following cuticle isolation (426–518 µg cm^−2^ depending on the cultivar). The nectarine cuticular wax contents obtained here are very close to those obtained for cherry, another glabrous stone fruit [20–48 µg cm^−2^ at maturity, according to Belge *et al.* (2014a) and Peschel *et al.* (2007)]. In fact, the general trends in the wax composition reported in these different studies are similar. As for peaches and cherries, free triterpenoid acids were the predominant surface compounds on nectarines, accounting for approximately 85% of the waxes on the fruit of both cultivars at maturity (Table 2). Since triterpenoids are located almost exclusively in the intracuticular wax layer of the leaves (Jetter and Schaffer, 2001; Buschhaus and Jetter, 2011) and fruit (Vogg *et al.*, 2004; Wang *et al.*, 2014), our results suggest that the chloroform dipping procedure we used, although probably not exhaustive, yielded waxes from both the epicuticular and intracuticular layers covering the fruit surface.

The primary free triterpenoids in the nectarine waxes were ursolic and oleanolic acids, with an ursolic/oleanolic acids ratio ranging from 2 to 3, depending on the cultivar (Fig. 5, Supplementary Table S1). These isomeric compounds were previously identified at maturity in peaches (Belge *et al.*, 2014b; Belge *et al.*, 2019). They are also predominant in the fruit cuticular waxes of other rosaceous fruit (Lara *et al.*, 2015). In addition to these major compounds, we quantified two dihydroxylated derivatives of ursolic or oleanolic acids that could be two of the triterpenoids previously reported in the peels of unripe nectarines (El Lahlou *et al.*, 1999). These derivatives constituted a significant proportion of the free triterpenoids at maturity, from 21% to 34% depending on the cultivar (Fig. 5, Supplementary Table S1). Additionally, we detected small amounts of monohydroxylated derivatives of ursolic and oleanolic acids that we tentatively identified as corosolic and maslinic acids, respectively (Fig. 2, Table 1), which were previously identified in the hulls of almonds (*Prunus dulcis*), a close relative of peach (Amico *et al.*, 2006).

HPLC-DAD analyses of nectarine waxes allowed us to detect new compounds identified as hydroxycinnamate esters of triterpenoids acids or of sugars (Fig. 2, Table 1). The hydroxycinnamate esters of triterpenoid acids were primarily acylated with *p*-coumaric acid, but a feruloyl derivative was also detected. *p*-Coumaroyl-triterpenoids constituted approximately 1% of the wax compounds at maturity (Table 2). Similar hydroxycinnamate esters of triterpenoids have been detected in apple (McGhie *et al.*, 2012) and pear peels (Serra *et al.*, 2018). To the best of our knowledge, this is the first report of the presence of these compounds in the cuticular waxes of rosaceous fruits.

*p*-Coumaroyl-acetylsugars were found only at the very beginning of nectarine fruit development (Fig. 3, Supplementary Fig. S2, Table 2). Their dihexoside moiety, which was acylated with five acetyl groups, was not further characterized. Related compounds (i.e. acetylhexosides esterified with *p*-coumaric acid) have been previously detected in the leaves and stems of bilberry (Bujor *et al.*, 2016). To the best of our knowledge, this is the first time these compounds have been found in fruit cuticular waxes.

At fruit maturity, alkanes constituted approximately 9% of the nectarine cuticular waxes and represented the major class of lipids beside free triterpenoids (Table 2), as previously described for peaches, in which they reached 17–29% of the wax content depending on the cultivar (Belge *et al.*, 2014b). As in peaches, odd-numbered very-long-chain alkanes ranging from C23 to C29 were the most important compounds in the nectarine waxes, whereas C31 hentriacontane, often a major alkane of fruit cuticular waxes (Lara *et al.*, 2015), was present in lower amounts (Supplementary Table S1).

Fatty acids accounted for 2.8–3.8% of the wax compounds at maturity (Table 2), less than reported values in peaches (8–10%; Belge *et al.*, 2014b). C22 to C28 very-long-chain fatty acids have not been reported in peaches, and they predominated among the fatty acid composition of waxes during the later stages of nectarine development (Supplementary Table S1).

As presumably derived from very-long-chain fatty acids, fatty alcohols and fatty aldehydes were also represented by even-numbered very-long-chain compounds at fruit maturity (Supplementary Table S1). Fatty alcohols represented 1.7–1.9% of the wax content (Table 2), slightly less than that reported in peaches (2.6%; Belge *et al.*, 2014b). Although C23 tricosanol was the primary compound in peaches, it was a minor constituent of nectarine waxes, in which the C24 to C30 compounds predominated. We also detected small amounts of fatty aldehydes, which have not been described in peaches, and which could be the intermediates in alkane biosynthesis from fatty acids. These compounds made up 0.4% of the wax contents, and were primarily represented by C30 triacontanal (Table 2, Supplementary Table S1).

### Remarkable changes in wax deposition during nectarine fruit development

Little is known about the dynamics of cuticular wax deposition during rosaceous stone fruit development (Lara *et al.*, 2015). Our study of this process in nectarines showed that cuticular waxes greatly varied both quantitatively and qualitatively throughout fruit growth. The wax load was very low on very young fruit and increased sharply during stage I (Fig. 5, Supplementary Table S1). This increase was primarily due to the heavy deposition of ursolic and oleanolic acids, which ceased in the middle of stage II (Fig. 3). Phenolic derivatives of triterpenoids, which remained quantitatively minor compounds, accumulated in a similar way but with a delay, accumulating primarily at the beginning of stage II (Fig. 3, Supplementary Fig. S2). This delay was particularly marked in 2015. It is noteworthy that previous studies on cherries showed a similar pattern for the formation of the whole cuticle, with a maximum rate of deposition occurring during stage I of fruit development followed by a rapid decline during stages II and III (Alkio et al., 2012, 2014). This finding suggests that for these two stone fruits, an early and very active phase of cuticle formation takes place during the first rapid growth of the fruit. It would include both the deposition of triterpenoid waxes and the formation of a cutin polymer on the skin of very young fruit, which is initially almost entirely devoid of a protective covering. In cherries, the expression of 13 genes that are possibly involved in cuticle formation in the exocarp was positively correlated with the rate of cuticle deposition (Alkio *et al.*, 2012). However, no expression of genes potentially involved in the triterpenoid biosynthetic process was reported in cherries during this phase (Alkio et al., 2012, 2014). Like cuticle deposition in cherries, the accumulation of cuticular waxes in nectarines ceased early in the fruit development process, during endocarp lignification before growth resumes in stage III (Fig. 5). At the middle of stage II, the triterpenoid contents decreased, both per unit surface area (Figs 3 and 5) and per fruit (Supplementary Fig. S4), indicating not only a dilution effect due to fruit growth but also a probable degradation or leaching of these compounds. A clear decrease in the total amount of waxes per fruit was also observed (Supplementary Fig. S3). This discrepancy between the deposition dynamics of cuticular wax and fruit growth may generate strain in the cuticle, which could be the origin of microcracks in the skin of the fruits of these two species that arise as maturity approaches (Peschel and Knoche, 2005; Gibert *et al.*, 2007). However, in nectarines, the total wax content increased again, with alkanes accumulating heavily from the beginning of the stage III of growth followed by a new increase in trihydroxylated triterpenoids that was observed only in 2012 (Figs 4 and 5B). In cherries, Peschel *et al.* (2007) observed that the deposition of alkanes and fatty alcohols kept pace with the surface expansion but the deposition of triterpenoids did not, resulting in a gradually higher proportion of alkanes relative to triterpenoids during stage III. This increasing proportion of alkanes may play a role in protecting fruit from cracking because high alkane levels have been associated with less fruit cracking in cherries (Rios *et al.*, 2015). The timing of the deposition of the different classes of cuticular wax compounds on the nectarine surface is summarized in Supplementary Fig. S3.

### Variation within cultivars and between years

While no obvious differences were observed between cultivars, the variations between years were more pronounced, with the free triterpenoids being quite high in 2015 compared with 2012 (Fig. 3). These variations could be explained by changes in environmental conditions during the primary period of wax deposition in early spring, when weather conditions are highly variable in the Mediterranean climate. It is well established that light and temperature as well as drought can affect fruit cuticular waxes (Trivedi *et al.*, 2019). Lv *et al.* (2016) showed that the contents of oleanolic and ursolic acids on the shaded side of apples were higher than those on the sun-exposed side. In pear peels, the levels of the different triterpene groups showed significant and contrasting variations depending on the fruit position in the canopy (Serra *et al.*, 2018).

### Role of cuticular waxes in preventing fruit gas exchange

Our study indicated that the highest surface conductance of nectarines was associated with a very low load of cuticular waxes on the surface of very young fruit (Fig. 6). The conductance dropped dramatically when triterpenoids were deposited, before increasing slightly again until maturity as these compounds decreased (Figs 1 and 3). In nectarine fruits, variations in surface conductance may result from the combined effects of the presence of stomata, characteristics of the cuticle, and the occurrence of cuticular cracks (Gibert *et al.*, 2010). However, no relationship was found between the total fruit surface conductance and the stomatal density (Gibert *et al.*, 2010). Thus, it is tempting to assign a major role to the cuticular composition in the control of water exchange in developing fruit. Although it is well established that wax removal greatly increases cuticular permeability, clear conclusions cannot be drawn from the literature about the role of the cuticle thickness or the wax load on the transpiration rate of organs. In plant organs from 61 species, including leaves and (in the few species studied) fruit, Riederer and Schreiber (2001) found no correlation between the cuticular water permeability and the thickness of the cuticle or wax coverage. Conversely, in tomato fruits, Romero and Rose (2019) suggested a relationship between the cuticle thickness or load and the transpiration rate. In fact, the organization and composition of cuticular waxes, rather than their quantity, are critical determinants of cuticle permeability. In a report on the desert plant *Rhazya stricta,* in which the leaf cuticular waxes are primarily composed of pentacyclic triterpenoids with a small proportion of long-chain aliphatics, as in nectarines, Schuster *et al.* (2016) argue that triterpenoid deposition strengthens the cutin matrix, protecting this polymer against elevated temperatures and preserving its efficacy as a transpiration barrier. Further investigations on the deposition process of triterpenoids in nectarines may provide a better understanding of their role as a potential barrier to transpiration in the cuticle.

### Potential role of cuticular waxes in brown rot infection

The susceptibility of nectarine fruits to brown rot infection changed greatly during their development, with two phases of high susceptibility at the beginning of the growth of very young fruit and at maturity, interrupted by an intermediate phase of high resistance during endocarp lignification (Fig. 1). This pattern of change was observed first by Biggs and Northover (1988) in peach fruits and was confirmed in peaches and apricots by Mari *et al.* (2003) and more recently by Guidarelli *et al.* (2014) in peaches. Fourie and Holz (2003) showed that nectarine fruits at the pit hardening stage were resistant to penetration by and development of *M. laxa*. In the present study, we observed the same dynamics in terms of disease susceptibility. Whereas fruits were highly susceptible to the fungus during stage I, they became remarkably resistant to *M. laxa* within a few days at the beginning of stage II (Fig. 1). This well-described dramatic change in susceptibility has led to research on changes in the composition (Bostock *et al.*, 1999; Villarino *et al.*, 2011) and gene expression (Guidarelli *et al.*, 2014) of peach skin at susceptible and resistant stages. These earlier studies focused on the metabolism of phenylpropanoids, particularly caffeic acid derivatives such as chlorogenic acid and its isomers. According to these authors, phenolic compounds in the epidermis, which were present at especially high concentrations in immature fruit but had no direct effect on spore germination and mycelium growth, could play a role in *Monilinia* resistance by suppressing the expression of fungal cutinases and polygalacturonases (Bostock *et al.*, 1999; Lee and Bostock, 2007) or by inhibiting the fungus’s production of melanin-like compounds, which could play a role in its pathogenicity (Villarino *et al.*, 2011). 

The cuticle, the outermost layer covering the fruit, not only forms a mechanical barrier, but also plays a significant and active role in plant–pathogen interactions (Serrano *et al.*, 2014; Ziv *et al.*, 2018). In Asian pear fruits, cuticular waxes inhibited conidial germination and mycelial growth of *Alternaria alternata* (Yin *et al.*, 2011), and changes in cuticular waxes during fruit development have been associated with variations in fruit susceptibility to the fungus (Li *et al.*, 2014). The role of cuticular waxes in the resistance of stone fruit to *Monilinia* has not been studied to date. In this study on nectarines, we observed a clear negative relationship between the amounts of cuticular wax components, especially the primary triterpenoids ursolic and olenolic acids, and susceptibility to brown rot (Fig. 7). We particularly noticed the coincidence of the heavy deposition of free triterpenoids and the establishment of resistance to *M. laxa* at the onset of stage II. Ursolic and oleanolic acids are well known to have broad antimicrobial properties (Jesus *et al.*, 2015), and these compounds and their derivatives have been characterized for their antifungal activity (Shai *et al.*, 2008). In nectarines, El Lahlou *et al.* (1999) found that all the triterpenoids isolated from the peels of unripe fruit showed antifungal activity. Therefore, triterpenoids represent good candidates to explain the high resistance of fruit during endocarp lignification. Moreover, the decreased triterpenoid levels during stage III could partly explain the loss of fruit resistance to the fungus. Accordingly, microscopic observations of the fruit surface after spore deposition at the three developmental stages showed important spore germination at stages I and III and latent spores at stage II (Supplementary Fig. S5). However, the total wax and the triterpenoid contents did not correlate well with *Monilinia* susceptibility at maturity (all cultivars in both years of the study) or close to maturity (Magique in 2015) (Fig. 7). One reason for this observation could be the appearance of microcracks (Gibert *et al.*, 2009), offering an ‘open gate’ for the entry of the fungus, which could bypass the cuticular protection. Many studies have shown links between fruit cracking and the incidence of brown rot (see Lino *et al.*, 2016), suggesting that resistance factors are no longer effective when the cuticle loses its integrity. Since large fruit size and rapid fruit growth promote microcrack occurrence (Gibert *et al.*, 2007), this may also explain why the large and early Magique fruit from harvest 4 were highly susceptible to *Monilinia* despite the level of triterpenoids in their cuticular wax. Microscopic observations of the surface of fruit from the three cultivars confirmed the formation of a dense network of microcracks on the surface of mature fruit and preferential spore germination inside the cracks (Supplementary Fig. S6). Another reason for the high susceptibility at maturity could be the higher level in cuticular waxes of alkanes, which could favour the growth of the fungus, since they increased in content during the final growth stage until ripening. Fungi can use alkanes as a carbon source, and these aliphatic compounds enhanced the growth *in vitro* of *Botrytis cinerea* (Silva-Moreno *et al.*, 2016). In the same way, the overexpression in Arabidopsis of the *ECERIFERUM1* gene, which promotes alkane biosynthesis, increased the plant’s susceptibility to *Sclerotinia* (Bourdenx *et al.*, 2011).

Likewise, in very young fruit, we observed a positive relationship between *p*-coumaroyl-acetylsugars and brown rot susceptibility (Fig. 7). The involvement of these compounds in plant–fungal interactions remains unknown. They may act as positive signals for *Monilinia* development or solely be a biochemical marker of this susceptible state. Further investigations using *in vitro* bioassays of isolated compounds are needed to assess the impact of peach cuticular waxes on *Monilinia* development. As a priority, the effect of oleanolic and ursolic acids on *M. laxa* germination and hyphal growth could be tested on agar plates. Similarly, cuticular extracts from fruits at stage I and stage II could be compared for their inhibitory properties towards *Monilinia*.

In conclusion, this study provides new information on the composition of cuticular waxes in nectarines and on their changes during fruit development in relation to the fruit surface conductance and susceptibility to the brown rot pathogen *Monilinia*. Our results reveal the presence of new phenolic derivatives of ursolic and oleanolic acids and acetylated sugars in waxes, and they highlight the important deposition of triterpenoids at the beginning of the endocarp lignification stage. The striking changes in cuticular waxes may explain the observed variations in fruit conductance and susceptibility to *Monilinia*. Our results also suggest that the regulation of wax deposition at the beginning of fruit development and the environmental factors that influence it are probably key points that determine the subsequent resistance of fruit to *Monilinia* and to water loss at harvest and during post-harvest conservation.

## Supplementary data

Supplementary data are available at *JXB* online.

**Table S1.** Wax compound contents from the surface of Summergrand and Zéphir nectarines during fruit development.

**Fig. S1.** Relationship between fruit fresh mass and surface conductance and infection probability.

**Fig S2.** Evolution of secondary compounds from nectarine cuticular waxes during fruit development analysed by HPLC.

**Fig S3.** Changes in total wax compounds per fruit from nectarine surface during fruit development.

**Fig S4.** Changes in 42 wax compounds per fruit from nectarine surface during fruit development.

**Fig S5.** Scanning electron microscopic images of the surface of nectarines inoculated with *M. laxa* spores at the three developmental stages.

**Fig S6.** Microcracks developing at stage III of nectarine development.

**Dataset S1.** Results of likelihood ratio tests to compare nested generalized linear mixed-effects models.

**Dataset S2.** Shape of the relationships between surface conductance or infection probability and compounds.

eraa284_suppl_Supplementary_Material
