# Supplementary material for: Cuticular waxes of nectarines during fruit development in relation to surface conductance and susceptibility to Monilinia laxa
Source: J Exp Bot. 2020 Jun 18;71(18):5521–37. doi: 10.1093/jxb/eraa284 (PMC7501825; doi:10.1093/jxb/eraa284)

Table S1.

Wax compound contents in µg.cm^{-2} (mean and standard deviation (*sd*)) from surface of Summergrand and Zéphir nectarines during fruit development (DAB: days after bloom) in 2012.

		Summergrand							Zéphir						
DAB		41	62	79	97	111	125	135	42	63	80	98	112	126	140
**Free triterpenoids**	mean	**8,39**	**42,76**	**63,25**	**38,07**	**19,89**	**18,1**	**26,62**	**10,79**	**48,37**	**96,23**	**51,21**	**25,62**	**30,92**	**35,07**
	sd	*0,19*	*16,14*	*7,42*	*3,64*	*5,84*	*1,48*	*4,16*	*0,86*	*11,1*	*28,55*	*6,75*	*1,98*	*4,04*	*3,75*
Ursolic acid (Urs)	mean	6,78	30,8	42,94	25,82	11,65	9,01	15,67	8,91	33,21	64,87	34,71	14,67	15,38	15,51
	sd	*0,13*	*13,22*	*5,42*	*3,44*	*3,49*	*0,9*	*2,32*	*0,79*	*7,87*	*20,29*	*7,67*	*1,4*	*2,33*	*1,28*
Oleanoic acid (Ole)	mean	1,61	8,28	15,74	9,5	4,68	4,1	5,47	1,88	10,65	24,44	14,52	7,6	8,17	7,56
	sd	*0,16*	*2,01*	*1,6*	*0,46*	*1,39*	*0,43*	*1,48*	*0,11*	*2,13*	*6,11*	*0,99*	*0,58*	*0,91*	*0,77*
Trihydroxy-urs-12-en-28-oic acid (thu1)	mean	0	1,39	2,15	1,34	1,62	2,28	2,65	0	1,9	3,49	1,04	1,77	3,76	6,13
	sd	*0*	*0,48*	*0,51*	*0,17*	*0,46*	*0,35*	*0,37*	*0*	*0,55*	*1,19*	*0,22*	*0,25*	*0,97*	*1,24*
Trihydroxy-urs-12-en-28-oic acid (thu2)	mean	0	2,29	2,42	1,41	1,94	2,71	2,83	0	2,61	3,43	0,94	1,58	3,61	5,87
	sd	*0*	*0,75*	*0,52*	*0,15*	*0,57*	*0,42*	*0,32*	*0*	*0,68*	*1,29*	*0,21*	*0,16*	*0,98*	*1,2*
***p*-coumaroyl-triterpenoids**	mean	**0**	**0,17**	**0,57**	**0,84**	**0,39**	**0,32**	**0,34**	**0**	**0,18**	**0,82**	**1,22**	**0,55**	**0,47**	**0,43**
	sd	*0*	*0,02*	*0,04*	*0,1*	*0,13*	*0,04*	*0,06*	*0*	*0,03*	*0,04*	*0,35*	*0,09*	*0,15*	*0,05*
p-coumaroyl-2,3-dihydroxy-urs-12-en-28-oic acid (cdhu3)	mean	0	0,02	0,13	0,15	0,08	0,06	0,08	0	0,03	0,22	0,26	0,11	0,11	0,11
	sd	*0*	*0*	*0,02*	*0,03*	*0,03*	*0,01*	*0,01*	*0*	*0,01*	*0,02*	*0,07*	*0,03*	*0,06*	*0,01*
p-coumaroyl-2,3-dihydroxy-urs-12-en-28-oic acid (cdhu5)	mean	0	0,02	0,08	0,18	0,1	0,08	0,08	0	0,02	0,12	0,29	0,15	0,12	0,1
	sd	*0*	*0*	*0*	*0,01*	*0,03*	*0,01*	*0,02*	*0*	*0*	*0,01*	*0,1*	*0,03*	*0,03*	*0,01*
p-coumaroyl-2,3-dihydroxy-urs-12-en-28-oic acid (cdhu6)	mean	0	0,03	0,13	0,24	0,13	0,11	0,11	0	0,03	0,19	0,34	0,18	0,15	0,15
	sd	*0*	*0,01*	*0,02*	*0,03*	*0,04*	*0,02*	*0,02*	*0*	*0*	*0,03*	*0,12*	*0,03*	*0,03*	*0,02*
3β-*p*-coumaroyloxy-urs-12-en-28-oic acid (cou4)	mean	0	0,1	0,23	0,26	0,09	0,07	0,07	0	0,11	0,29	0,34	0,11	0,08	0,06
	sd	*0*	*0,01*	*0,01*	*0,06*	*0,03*	*0,02*	*0,01*	*0*	*0,01*	*0,02*	*0,09*	*0,02*	*0,04*	*0,01*
***p*-coumaroyl-acetylsugars**	mean	**0,29**	**0,03**	**0**	**0**	**0**	**0**	**0**	**0,15**	**0,02**	**0**	**0**	**0**	**0**	**0**
	sd	*0,07*	*0,01*	*0*	*0*	*0*	*0*	*0*	*0,01*	*0,01*	*0*	*0*	*0*	*0*	*0*
p-coumaroylpentacetyldihexoside (cpdh1)	mean	0,07	0,01	0	0	0	0	0	0,02	0,01	0	0	0	0	0
	sd	*0,01*	*0*	*0*	*0*	*0*	*0*	*0*	*0*	*0*	*0*	*0*	*0*	*0*	*0*
p-coumaroylpentacetyldihexoside (cpdh2)	mean	0,22	0,02	0	0	0	0	0	0,13	0,01	0	0	0	0	0
	sd	*0,06*	*0*	*0*	*0*	*0*	*0*	*0*	*0,01*	*0,01*	*0*	*0*	*0*	*0*	*0*
**Alkanes**	mean	**0,49**	**0,07**	**0,22**	**0,35**	**1,82**	**2**	**2,71**	**0,43**	**0,12**	**0,71**	**0,54**	**1,25**	**2,35**	**3,88**
	sd	*0,11*	*0,02*	*0,02*	*0,04*	*0,13*	*0,27*	*0,25*	*0,08*	*0,02*	*0,2*	*0,15*	*0,12*	*0,35*	*0,66*
C19 nonadecane (C19Alk)	mean	0	0	0,02	0,02	0,03	0,01	0,01	0	0	0,01	0,01	0,01	0,01	0,01
	sd	*0*	*0*	*0,01*	*0,01*	*0*	*0,01*	*0*	*0*	*0*	*0*	*0,01*	*0*	*0*	*0*
C20 eicosane (C20Alk)	mean	0,11	0	0	0	0	0	0	0,04	0	0	0	0	0	0
	sd	*0,03*	*0*	*0*	*0*	*0*	*0*	*0*	*0*	*0*	*0*	*0*	*0*	*0*	*0*
C21 henicosane (C21Alk)	mean	0,03	0,01	0,01	0,01	0,01	0,01	0,02	0,02	0,01	0,01	0,01	0	0,01	0,02
	sd	*0,02*	*0*	*0*	*0*	*0*	*0*	*0,01*	*0,01*	*0*	*0,01*	*0,01*	*0*	*0*	*0*
C23 tricosane (C23Alk)	mean	0,02	0,01	0,01	0,02	0,12	0,24	0,46	0,02	0,01	0,08	0,04	0,09	0,27	0,65
	sd	*0,01*	*0*	*0*	*0*	*0,01*	*0,04*	*0,11*	*0*	*0*	*0,04*	*0,02*	*0,01*	*0,07*	*0,16*
C24 tetracosane (C24Alk)	mean	0,05	0	0,01	0,02	0,03	0,03	0,05	0,02	0,01	0,02	0,02	0,02	0,04	0,06
	sd	*0,01*	*0*	*0*	*0*	*0*	*0,01*	*0,02*	*0,01*	*0*	*0*	*0,01*	*0*	*0,01*	*0,01*
C25 pentacosane (C25Alk)	mean	0,12	0,03	0,07	0,12	0,43	0,47	0,71	0,15	0,05	0,29	0,21	0,35	0,66	1,05
	sd	*0,06*	*0,01*	*0,01*	*0,02*	*0,03*	*0,06*	*0,09*	*0,07*	*0,01*	*0,1*	*0,07*	*0,06*	*0,13*	*0,2*
C27 heptacosane (C27Alk)	mean	0,11	0,01	0,03	0,04	0,44	0,48	0,62	0,13	0,02	0,03	0,04	0,38	0,62	0,97
	sd	*0,03*	*0,01*	*0,02*	*0,01*	*0,03*	*0,07*	*0,04*	*0,02*	*0,01*	*0,01*	*0,02*	*0,03*	*0,09*	*0,18*
C29 nonacosane (C29Alk)	mean	0,03	0,01	0,05	0,09	0,59	0,66	0,72	0,03	0,02	0,22	0,17	0,35	0,66	1,01
	sd	*0,01*	*0*	*0,01*	*0,02*	*0,04*	*0,11*	*0,08*	*0*	*0*	*0,05*	*0,05*	*0,02*	*0,08*	*0,15*
C31 hentriacontane (C31Alk)	mean	0,02	0	0,02	0,03	0,17	0,11	0,12	0,03	0,01	0,04	0,03	0,05	0,09	0,1
	sd	*0,01*	*0*	*0,01*	*0,01*	*0,01*	*0,01*	*0,01*	*0,01*	*0*	*0,02*	*0,01*	*0,01*	*0,01*	*0,02*
**Fatty acids**	mean	**0,25**	**0,13**	**0,5**	**0,49**	**0,85**	**1,08**	**0,7**	**0,09**	**0,31**	**0,79**	**0,74**	**0,31**	**0,69**	**1,58**
	sd	*0,07*	*0,04*	*0,17*	*0,11*	*0,06*	*0,39*	*0,24*	*0,01*	*0,1*	*0,13*	*0,18*	*0,11*	*0,09*	*0,5*
C16 hexadecanoic acid, Palmitic acid (C16FA)	mean	0,06	0,04	0,16	0,1	0,16	0,13	0,08	0,04	0,1	0,13	0,13	0,05	0,09	0,16
	sd	*0,01*	*0,01*	*0,06*	*0,02*	*0,01*	*0,04*	*0,03*	*0,01*	*0,04*	*0,03*	*0,04*	*0,01*	*0,02*	*0,07*
C18:0 octadecanoic acid, Stearic acid (C18FA)	mean	0,04	0,02	0,06	0,05	0,12	0,08	0,05	0,01	0,04	0,07	0,07	0,02	0,04	0,08
	sd	*0,01*	*0,01*	*0,03*	*0,01*	*0,01*	*0,04*	*0,02*	*0,01*	*0,02*	*0,01*	*0,02*	*0,01*	*0,01*	*0,03*
C18:1 oleic acid (C18_1FA)	mean	0,02	0	0,01	0,01	0,02	0,03	0,04	0,02	0,01	0,01	0,01	0,01	0,02	0,05
	sd	*0,01*	*0*	*0*	*0,01*	*0*	*0,01*	*0,02*	*0*	*0*	*0*	*0,01*	*0*	*0*	*0,02*
C18:2 linoleic acid (C18_2FA)	mean	0,05	0,02	0,09	0,09	0,18	0,21	0,06	0,02	0,05	0,14	0,1	0,05	0,11	0,13
	sd	*0,02*	*0,01*	*0,03*	*0,01*	*0,01*	*0,09*	*0,02*	*0*	*0,02*	*0,02*	*0,03*	*0,01*	*0,05*	*0,07*
C20 eicosanoic acid - Arachidic acid (C20FA)	mean	0,02	0	0,01	0,02	0,02	0,03	0,01	0	0,01	0,02	0,02	0	0,01	0,04
	sd	*0,01*	*0*	*0*	*0*	*0*	*0,02*	*0,01*	*0*	*0*	*0*	*0*	*0*	*0*	*0,02*
C22 docosanoic acid - Behenic acid (C22FA)	mean	0,03	0,02	0,07	0,11	0,04	0,03	0,13	0	0,04	0,27	0,22	0,01	0,05	0,14
	sd	*0,01*	*0,01*	*0,01*	*0,05*	*0*	*0,02*	*0,07*	*0*	*0,01*	*0,06*	*0,06*	*0,01*	*0,04*	*0,1*
C24 tetracosanoic acid - Lignoceric acid (C24FA)	mean	0,02	0,01	0,04	0,03	0,15	0,24	0,15	0	0,03	0,05	0,05	0,05	0,16	0,42
	sd	*0,01*	*0*	*0,03*	*0,01*	*0,01*	*0,1*	*0,05*	*0*	*0,01*	*0,02*	*0,02*	*0,02*	*0,02*	*0,15*
C26 hexanocosanoic acid - Cerotic acid (C26FA)	mean	0	0	0,03	0,05	0,15	0,21	0,13	0	0,01	0,07	0,1	0,07	0,15	0,41
	sd	*0*	*0*	*0*	*0,03*	*0,01*	*0,08*	*0,03*	*0*	*0,01*	*0,02*	*0,03*	*0,02*	*0,07*	*0,14*
C28 octacosanoic acid - Montanic acid (C28FA)	mean	0	0,01	0,03	0,03	0,02	0,12	0,03	0	0,02	0,02	0,04	0,04	0,05	0,16
	sd	*0*	*0*	*0,01*	*0,01*	*0*	*0,05*	*0,01*	*0*	*0,01*	*0*	*0,02*	*0,03*	*0,02*	*0,09*
**Fatty alcohols**	mean	**0,23**	**0,13**	**0,44**	**0,99**	**0,47**	**0,43**	**0,52**	**0,2**	**0,3**	**0,65**	**1,15**	**0,53**	**0,53**	**0,79**
	sd	*0,07*	*0,03*	*0,07*	*0,09*	*0,03*	*0,11*	*0,04*	*0,03*	*0,05*	*0,09*	*0,29*	*0,09*	*0,07*	*0,11*
C18 octadecanol (C18OH)	mean	0,03	0,01	0,01	0,01	0,04	0,01	0,02	0,02	0,01	0,03	0,02	0,01	0,01	0,02
	sd	*0,02*	*0*	*0*	*0*	*0*	*0*	*0*	*0*	*0*	*0,01*	*0,01*	*0*	*0*	*0,01*
C20 eicosanol (C20OH)	mean	0,04	0	0,01	0,01	0,01	0,01	0,02	0	0,01	0,01	0,01	0,01	0,01	0,02
	sd	*0,01*	*0*	*0*	*0,01*	*0*	*0*	*0,01*	*0*	*0*	*0*	*0,01*	*0*	*0*	*0*
C22 docosanol (C22OH)	mean	0,03	0,01	0,02	0,09	0,01	0,02	0,02	0,05	0,02	0,02	0,05	0,01	0,02	0,03
	sd	*0,01*	*0*	*0*	*0,08*	*0*	*0,01*	*0,01*	*0,04*	*0*	*0,01*	*0,03*	*0,01*	*0*	*0,02*
C23 tricosanol (C23OH)	mean	0,05	0	0	0,01	0,01	0,01	0,04	0	0,01	0,01	0,01	0	0,01	0,04
	sd	*0,02*	*0*	*0*	*0*	*0*	*0*	*0,02*	*0*	*0*	*0*	*0*	*0*	*0*	*0,01*
C24 tetracosanol (C24OH)	mean	0,08	0,02	0,04	0,07	0,03	0,05	0,09	0,12	0,04	0,05	0,09	0,05	0,05	0,11
	sd	*0,02*	*0,01*	*0,01*	*0,01*	*0*	*0,02*	*0,01*	*0,01*	*0,02*	*0,01*	*0,02*	*0,01*	*0,01*	*0,01*
C26 hexacosanol (C26OH)	mean	0	0,05	0,17	0,33	0,14	0,12	0,14	0	0,15	0,2	0,39	0,16	0,17	0,25
	sd	*0*	*0,02*	*0,02*	*0,04*	*0,01*	*0,04*	*0,01*	*0*	*0,03*	*0,04*	*0,1*	*0,03*	*0,03*	*0,03*
C28 octacosanol (C28OH)	mean	0	0,02	0,09	0,25	0,13	0,12	0,12	0	0,06	0,18	0,32	0,13	0,14	0,2
	sd	*0*	*0,01*	*0,03*	*0,03*	*0,01*	*0,03*	*0,01*	*0*	*0,01*	*0,02*	*0,07*	*0,02*	*0,02*	*0,02*
C30 triacontanol (C30OH)	mean	0	0,01	0,09	0,2	0,1	0,1	0,08	0	0,02	0,15	0,27	0,15	0,12	0,13
	sd	*0*	*0*	*0,01*	*0,03*	*0,01*	*0,02*	*0,01*	*0*	*0,01*	*0,02*	*0,06*	*0,03*	*0,02*	*0,01*
**Fatty aldehydes**	mean	**0**	**0,01**	**0,11**	**0,11**	**0,13**	**0,12**	**0,13**	**0**	**0,02**	**0,11**	**0,17**	**0,1**	**0,13**	**0,17**
	sd	*0*	*0*	*0,04*	*0,03*	*0,01*	*0,03*	*0,04*	*0*	*0,01*	*0,01*	*0,05*	*0,02*	*0,03*	*0,04*
C18 octadecanal (C18Aldh)	mean	0	0	0,06	0,02	0	0,01	0,01	0	0	0,03	0,03	0,01	0	0,01
	sd	*0*	*0*	*0,03*	*0,01*	*0*	*0*	*0,01*	*0*	*0*	*0,01*	*0,01*	*0*	*0*	*0*
C22 docosanal (C22Aldh)	mean	0	0	0	0	0	0	0,01	0	0	0	0	0	0	0,01
	sd	*0*	*0*	*0*	*0*	*0*	*0,01*	*0*	*0*	*0*	*0*	*0*	*0*	*0*	*0*
C24 tetracosanal (C24Aldh)	mean	0	0	0	0	0,02	0,04	0,03	0	0	0	0	0,02	0,03	0,05
	sd	*0*	*0*	*0*	*0*	*0*	*0,01*	*0,01*	*0*	*0*	*0*	*0*	*0*	*0,01*	*0,01*
C30 triacontanal (C30Aldh)	mean	0	0,01	0,05	0,09	0,11	0,07	0,08	0	0,02	0,07	0,14	0,08	0,11	0,1
	sd	*0*	*0*	*0,03*	*0,02*	*0,01*	*0,03*	*0,03*	*0*	*0,01*	*0,01*	*0,04*	*0,01*	*0,03*	*0,03*
**Phytosterols**	mean	**0,27**	**0,07**	**0,39**	**0,42**	**0,2**	**0,19**	**0,21**	**0,4**	**0,18**	**0,43**	**0,51**	**0,11**	**0,14**	**0,19**
	sd	*0,09*	*0,02*	*0,05*	*0,04*	*0,01*	*0,07*	*0,05*	*0,07*	*0,04*	*0,23*	*0,14*	*0,02*	*0,02*	*0,03*
Stigmasta-3,5-diene	mean	0,19	0,01	0,03	0,02	0,08	0,03	0,05	0,32	0,01	0,04	0,04	0,02	0,03	0,04
	sd	*0,05*	*0*	*0,01*	*0,01*	*0,01*	*0*	*0,01*	*0,05*	*0*	*0,01*	*0,01*	*0,01*	*0,01*	*0*
β-Sitosterol	mean	0,08	0,06	0,36	0,4	0,11	0,15	0,16	0,08	0,16	0,4	0,47	0,09	0,11	0,15
	sd	*0,04*	*0,02*	*0,05*	*0,04*	*0,01*	*0,07*	*0,06*	*0,04*	*0,04*	*0,24*	*0,13*	*0,02*	*0,01*	*0,03*
**Total**	mean	**9,92**	**43,37**	**65,49**	**41,26**	**23,75**	**22,23**	**31,23**	**12,07**	**49,49**	**99,74**	**55,55**	**28,46**	**35,22**	**42,10**
	sd	*0,41*	*16,16*	*7,33*	*3,82*	*5,96*	*1,98*	*4,32*	*0,88*	*11,13*	*28,32*	*7,09*	*2,13*	*4,14*	*4,11*

Fig. S1.

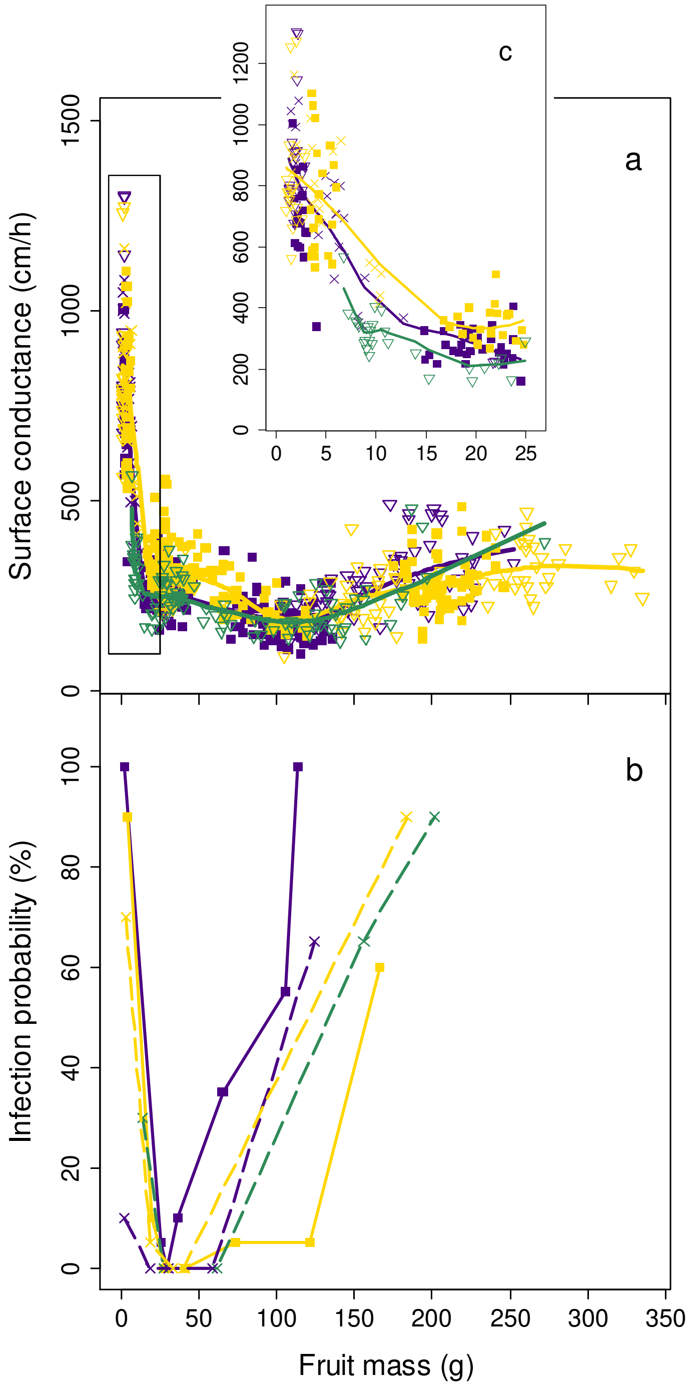

Relationship between fruit fresh mass and surface conductance (a, c) and infection probability (b). The graph c corresponds to the framed area of a. Full squares, crosses and empty triangles correspond to data obtained in 2012, 2015, and both 2013 and 2014 respectively, for three cultivars, Summergrand (indigo), Zéphir (gold) and Magique (green). Plain and longdash lines of plot b refer to 2012 and 2015 data respectively, for each cultivar.

Fig. S2

Evolution of secondary compounds from nectarine cuticular waxes during fruit development analyzed by HPLC. Squares and crosses correspond to 2012 and 2015 data respectively, from three cultivars, Summergrand (indigo), Zéphir (gold) and Magique (green) (complement to Fig. 3). They belong to three families: *p*-coumaroyl-acetylsugars (a, b), free triterpenoids (c, d) and *p*-coumaroyl-triterpenoids (e, f, g, h, i, j). Lines are fitted linear models by GLMM according to significant genotype and DAB (quadratic and cubic terms) effects. The fruit growth stages marked are those of Summergrand and Zéphir cultivars. See Table 1 for signification of

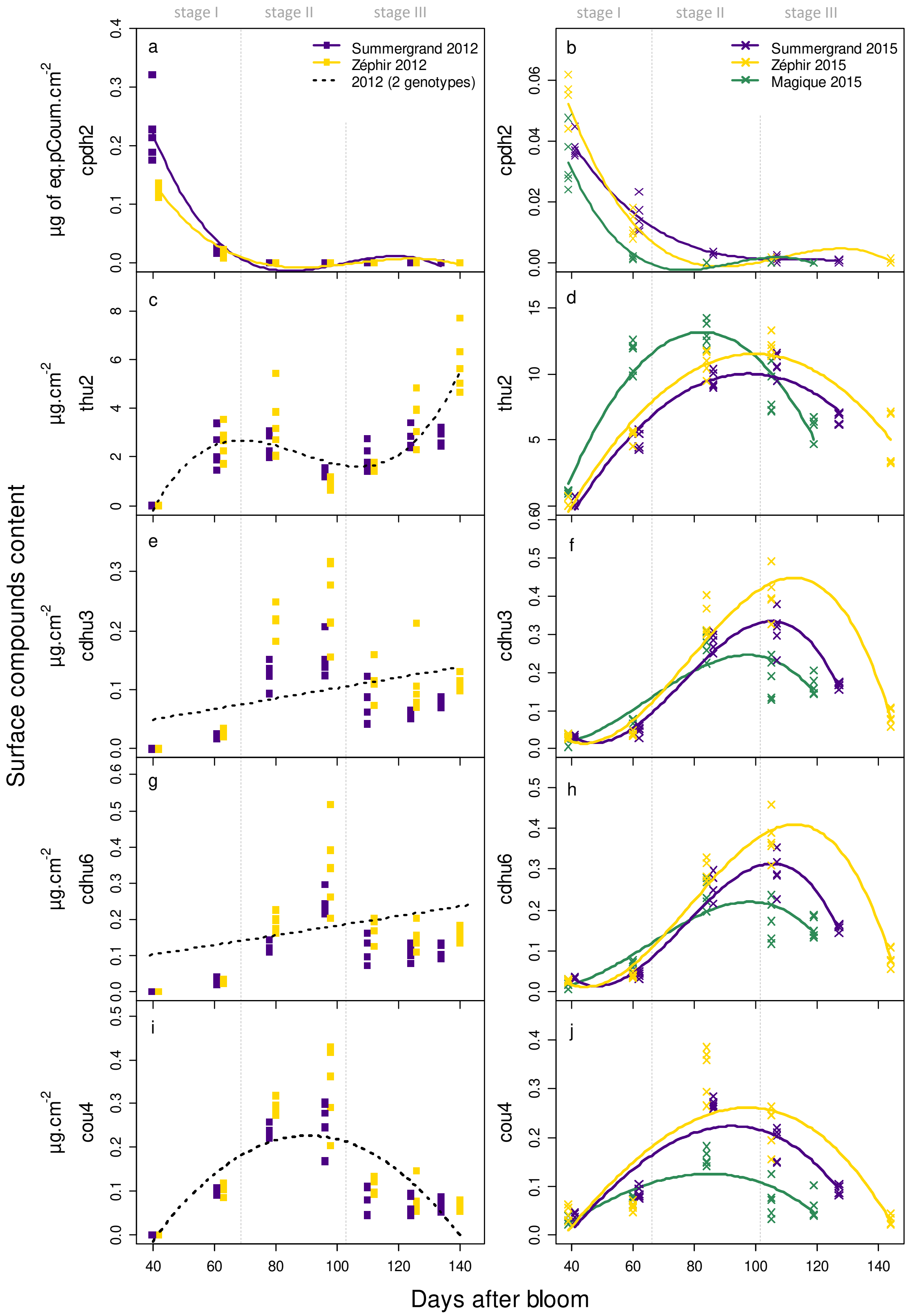

compound abbreviations and Supplementary Statistical results S1 for GLMM results.

Fig. S3.

Evolution in µg/fruit of total wax compounds from nectarine surface identified in Summergrand (indigo) and Zéphir (gold) during fruit development in 2012. The main deposition periods for the different classes of compounds are summarized. The black line is the fitted linear model by GLMM (no significant genotype effect). See Supplementary Statistical results S1 for GLMM results.

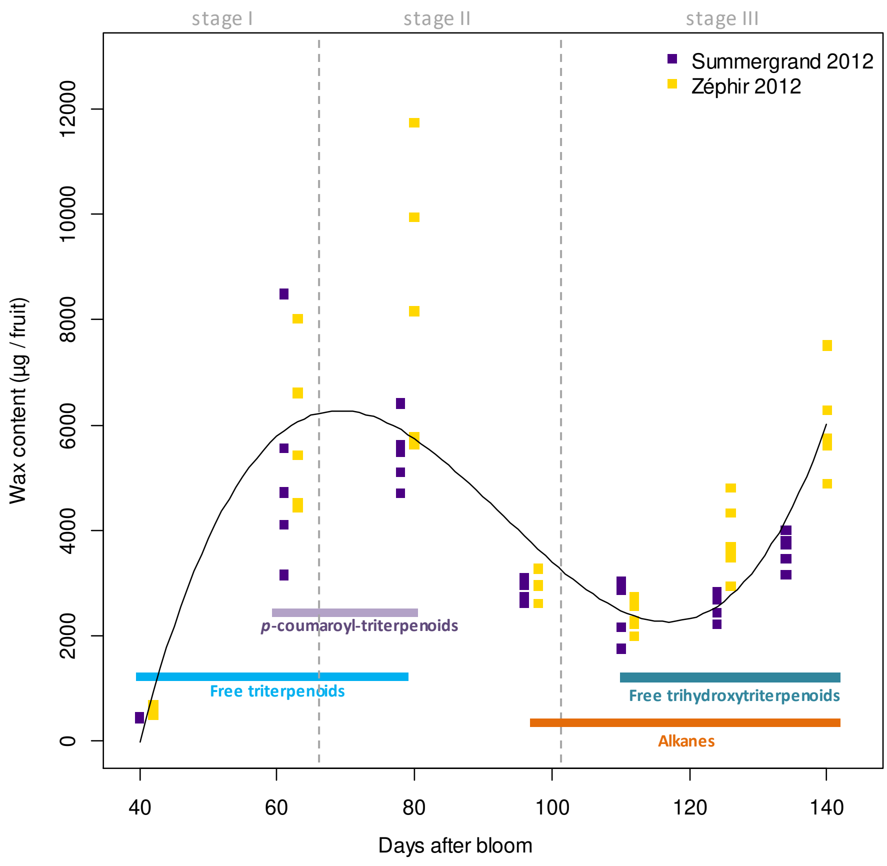

Fig. S4.

Evolution in µg/fruit of 42 wax compounds from nectarine surface identified in Summergrand and Zéphir during fruit development. Squares and crosses correspond to 2012 and 2015 data respectively. from three cultivars. Summergrand (indigo). Zéphir (gold) and Magique (green). Plain and dotted lines were obtained by smoothing 2012 and 2015 data respectively. Compound abbreviations given in Table S1.

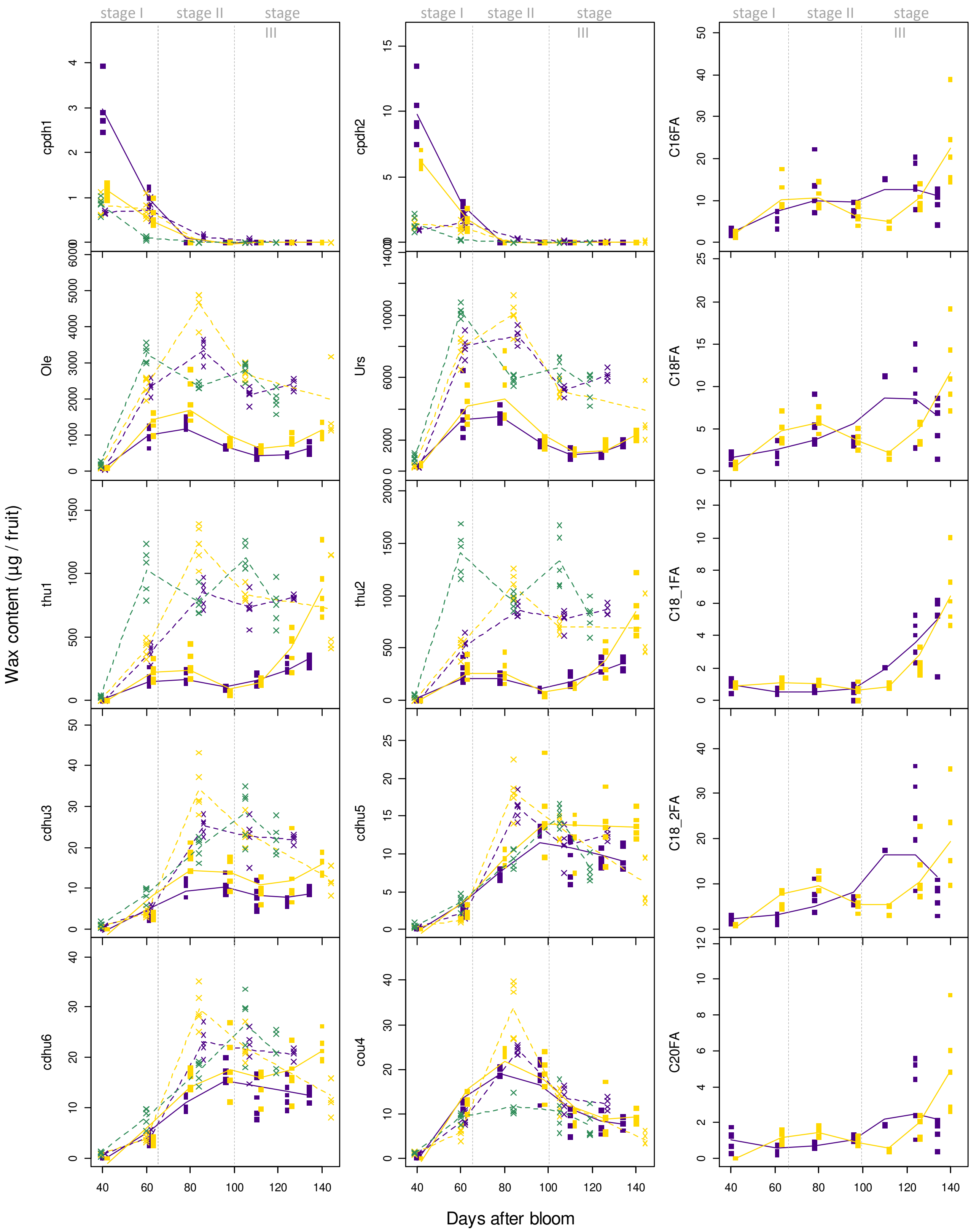

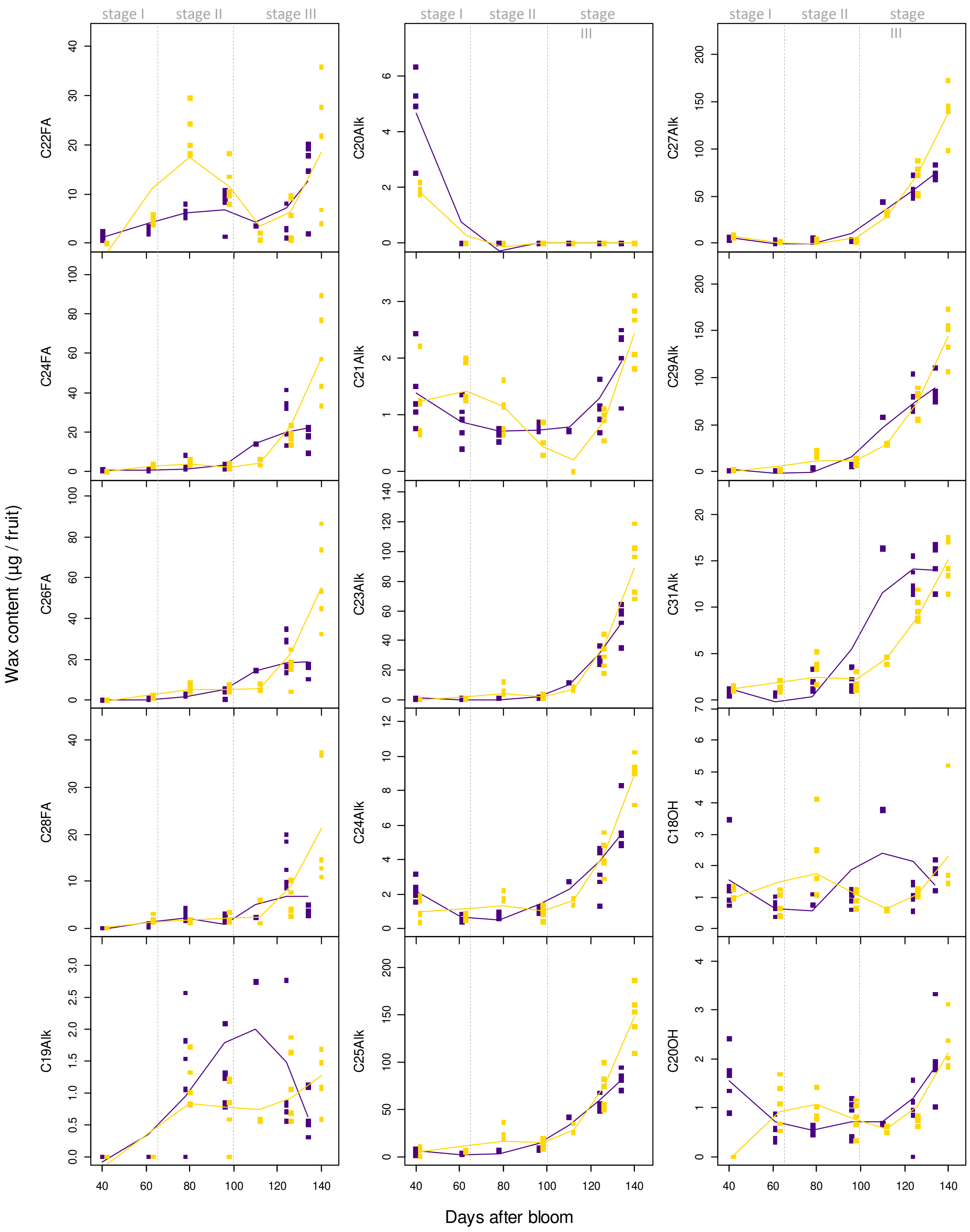
stage I
stage II
stage III
C22FA
C24FA
C26FA
C28FA
C19Alk
C20Alk
C21Alk
C23Alk
C24Alk
C25Alk
C27Alk
C29Alk
C31Alk
C18OH
C20OH
Wax content (µg / fruit)
Days after bloom
40
60
80
100
120
140

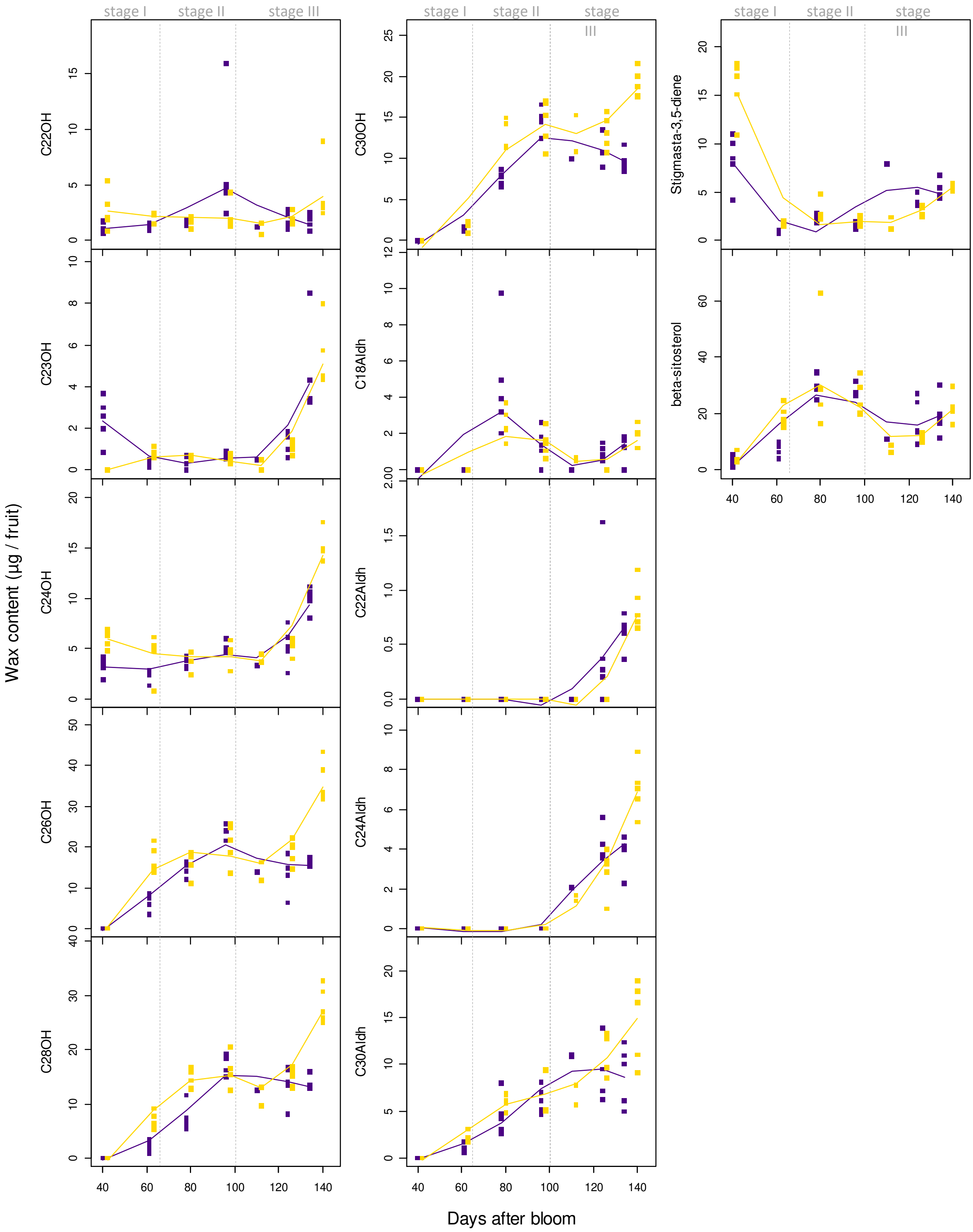
stage I
stage II
stage III
C22OH
C30OH
Stigmasta-3,5-diene
C23OH
C18Aldh
beta-sitosterol
C24OH
C22Aldh
C26OH
C24Aldh
C28OH
C30Aldh
Wax content (µg / fruit)
Days after bloom
40
60
80
100
120
140

Fig. S5.

Scanning Electron Microscopy images of the surface of nectarines inoculated with *M. laxa* spores at the three developmental stages:

A: Image showing the development of hyphae from *M. laxa* on a very young fruit surface (60 days after bloom, stage I), 5 hours after infection. Arrows point to a stoma (*s*) and hyphae (*h*).

B: Image showing Zéphir fruit surface at the end of stage II. Arrows point to non-germinating spores.

C: Image showing a very dense network of hyphae from *M. laxa* at the surface of mature fruit of Zéphir cultivar (stage III), 24 h after infection.

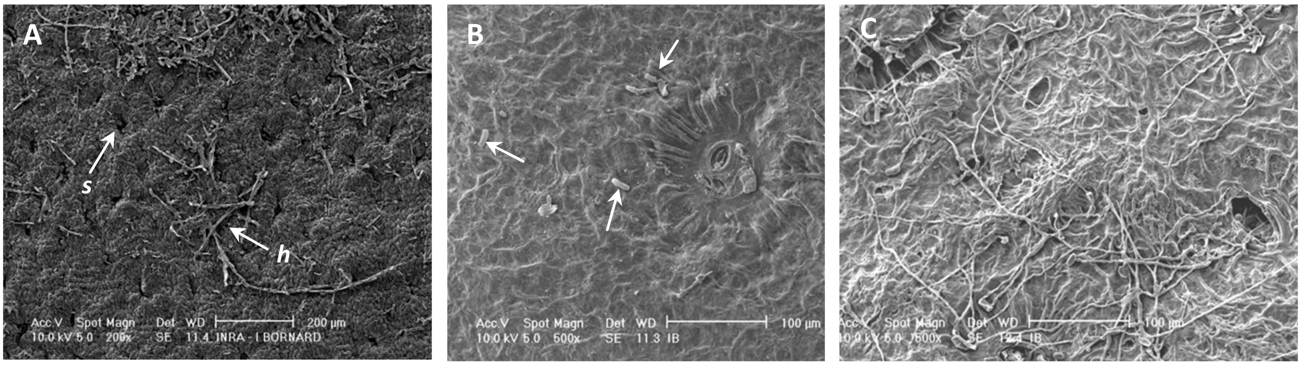

Fig. S6.

Microcracks developing at stage III of nectarine development:

A: Image showing the surface of a big Zéphir fruit at maturity with a dense network of microcracks (observed under stereo microscope). Cracks were stained dark blue by applying toluidine blue at 0.1%. Bar scale, 0.1 mm.

B: Scanning Electron Microscopy image showing spores germinating (arrows) in the microcracks of mature fruit of Summergrand cultivar (stage III).

C: Scanning Electron Microscopy image showing the development of hyphae from *M. laxa* in microcracks on the surface of mature fruit of Magique cultivar (stage III). Arrows point to a lenticel (presumably derived from a stoma which is no longer functional) (*l*), microcracks (*m*) and hyphae (*h*).

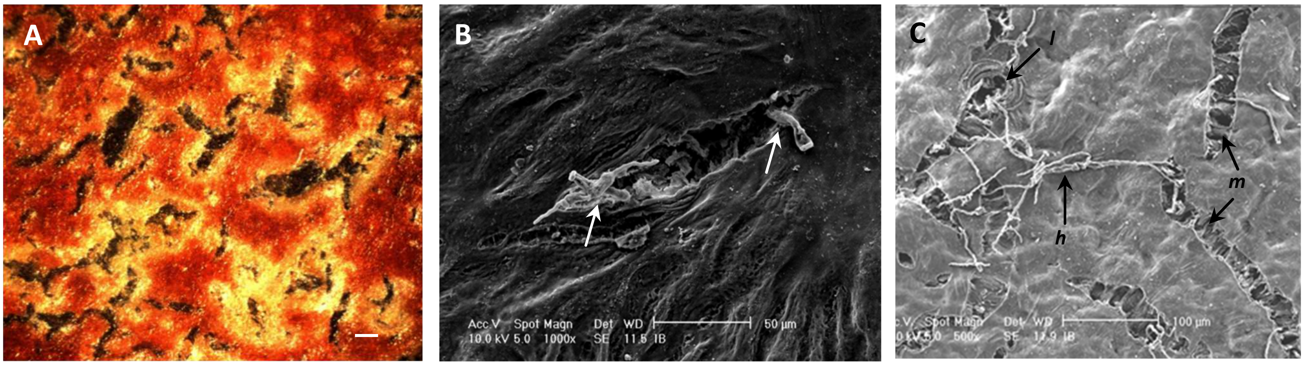

Statistical results S1.

Results of likelihood ratio tests to compare nested generalized linear mixed-effects models. The lmer and ANOVA functions in the 'lmer4' library were used. Repetitions were fruit or fruit lots harvested at one given date for each genotype.

Test of the effect of the genotype, the DAB, the quadratic and cubic terms of the DAB and the DAB:genotype interaction on the mass evolution of the 3 genotypes (Summergrand, Zéphir and Magique).

**Complete model**		**Df**	**AIC**	**logLik**			
mass ~ genotype + DAB + DAB^2+ DAB^3 + DAB: genotype + (1 | fruit)		10	4591.4	-2286			
**Tested model**	**Tested effect**				**Chisq**	**Chi Df**	**Pr(>Chisq)**
mass ~ DAB + DAB^2+ DAB^3 + DAB:genotype + (1 | fruit)	genotype	8	4916.8	-2450	329.44	2	< 2.2e-16 ***
mass ~ genotype + DAB:genotype + (1 | fruit)	DAB	8	5188.9	-2586	601.56	2	< 2.2e-16 ***
mass ~ genotype + DAB + DAB^3 + DAB: genotype + (1 | fruit)	DAB quadractic term	9	4651.4	-2317	62.03	1	3.379e-15 ***
mass ~ genotype + DAB + DAB^2 + DAB: genotype + (1 | fruit)	DAB cubic term	9	4699.8	-2341	110.46	1	< 2.2e-16 ***
mass ~ genotype + DAB + DAB^2+ DAB^3 + (1 | fruit)	DAB:genotype	8	5205.5	-2595	618.17	2	< 2.2e-16 ***

Test of the effect of the year and the year:DAB and year:genotype interactions on the mass evolution of 2 genotypes (Summergrand and Zéphir) for which year repetition is available.

**Complete model**		**Df**	**AIC**	**logLik**			
mass ~ year +genotype + DAB + DAB^2+ DAB^3 + year:genotype + year:DAB + DAB: genotype + (1 | fruit)		11	3580.8	-1779			
**Tested model**	**Tested effect**				**Chisq**	**Chi Df**	**Pr(>Chisq)**
mass ~ genotype + DAB + DAB^2+ DAB^3 + year:genotype + year:DAB + DAB:genotype + (1 | fruit)	year	10	3582.2	-1781	3.36	1	0.0666
mass ~ year +genotype + DAB + DAB^2+ DAB^3 + year:genotype + DAB: genotype + (1 | fruit)	year:DAB	10	3593.6	-1787	14.80	1	0.00012 ***
mass ~ year +genotype + DAB + DAB^2+ DAB^3 + year:DAB + DAB: genotype + (1 | fruit)	year:genotype	10	3606.7	-1793	27.87	1	1.299e-07 ***

Test of the effect of the genotype, the DAB, the DAB quadratic and cubic terms and the DAB:genotype interaction on the surface conductance evolution of 2 genotypes (Summergrand and Zéphir)

**Complete model**		**Df**	**AIC**	**logLik**			
conductance ~ genotype + DAB + DAB^2+ DAB^3 + DAB: genotype + (1 | fruit)		8	7321	-3652			
**Tested model**	**Tested effect**				**Chisq**	**Chi Df**	**Pr(>Chisq)**
conductance ~ DAB + DAB^2+ DAB^3 + DAB: genotype + (1 | fruit)	genotype	7	7319	-3653	0.77	1	0.38
conductance ~ genotype + DAB:genotype + (1 | fruit)	DAB	6	7845	-3916	528.5	2	< 2.2e-16 ***
conductance ~ genotype + DAB + DAB^3 + DAB: genotype + (1 | fruit)	DAB quadratic term		7531	-3759	212.9	1	< 2.2e-16 ***
conductance ~ genotype + DAB + DAB^2 + DAB: genotype + (1 | fruit)	DAB cubic term	7	7470	-3728	151.4	1	< 2.2e-16 ***
conductance ~ genotype + DAB + DAB^2+ DAB^3 + (1 | fruit)	DAB:genotype	7	7319	-3653	0.76	1	0.38

Test of the effect of the genotype, the DAB, the DAB quadratic and cubic terms and the DAB:genotype interaction on the total waxes (µg per fruit) evolution of 2 genotypes (Summergrand and Zéphir). on 2012 data. (See Figure S1)

**Complete model**		**Df**	**AIC**	**logLik**			
Total waxes ~ genotype + DAB + DAB^2+ DAB^3 + DAB: genotype + (1 | lot)		8	1211	-598			
**Tested model**	**Tested effect**				**Chisq**	**Chi Df**	**Pr(>Chisq)**
Total waxes ~ DAB + DAB^2+ DAB^3 + DAB:genotype + (1 | lot)	genotype	7	1209	-598	0.0018	1	0.966
Total waxes ~ genotype + DAB:genotype + (1 | lot)	DAB	6	1290	-639	82.35	2	< 2.2e-16 ***
Total waxes ~ DAB + DAB^3+ DAB:genotype + (1 | lot)	DAB quadratic term	7	1286	-636	77.09	1	< 2.2e-16 ***
Total waxes ~ DAB + DAB^2+ DAB:genotype + (1 | lot)	DAB cubic term	7	1283	-634	73.47	1	< 2.2e-16 ***
Total waxes ~ genotype + DAB + DAB^2+ DAB^3 + (1 | lot)	DAB:genotype	7	1210	-598	0.56	1	0.456

Summary of results (Chisq statistic and pvalue) for secondary compounds from nectarine cuticular waxes analyzed by HPLC for the two years (2012 and 2015) and the 3 genotypes (Summergrand, Zéphir and Magique). Tested effects by GLMM nested models (significance level of 0.01).

2012

**Tested effect**	**genotype**		**DAB**		**DAB quadratic term**		**DAB cubic term**		**DAB : gen**	
Variable	Chisq	Pr (>Chisq)	Chisq	Pr (>Chisq)	Chisq	Pr (>Chisq)	Chisq	Pr (>Chisq)	Chisq	Pr (>Chisq)
cpdh1	40.35	2.12e-10 ***	94.25	3.41e-21 ***	50.45	1.22e-12 ***	40.91	1.59e-10 ***	33.70	6.43e-09 ***
cpdh2	22.21	2.44e-06 ***	125.99	4.37e-28 ***	73.40	1.06e-17 ***	60.99	5.74e-15 ***	18.88	1.39e-05 ***
Ole	0.10	7.55e-01	72.07	2.24e-16 ***	34.13	5.15e-09 ***	26.76	2.30e-07 ***	1.10	2.95e-01
Urs	0.50	4.81e-01	71.27	3.34e-16 ***	42.79	6.11e-11 ***	35.92	2.05e-09 ***	0.00	9.58e-01
thu1	2.59	1.08e-01	49.07	2.21e-11 ***	46.03	1.16e-11 ***	47.72	4.92e-12 ***	8.33	3.90e-03 *
thu2	1.63	2.02e-01	59.34	1.30e-13 ***	57.69	3.07e-14 ***	58.90	1.66e-14 ***	3.77	5.23e-02
cdhu3	0.23	6.33e-01	42.27	6.63e-10 ***	0.38	5.39e-01	0.02	8.93e-01	3.16	7.57e-02
cdhu5	0.95	3.30e-01	45.08	1.62e-10 ***	5.72	1.68e-02	9.41	2.16e-03 *	4.46	3.47e-02
cdhu6	0.87	3.52e-01	42.95	4.71e-10 ***	1.44	2.30e-01	3.70	5.45e-02	4.05	4.43e-02
cou4	0.21	6.44e-01	75.15	4.79e-17 ***	8.74	3.11e-03 *	3.65	5.62e-02	1.27	2.60e-01

2015

**Tested effect**	**genotype**		**DAB**		**DAB quadratic term**		**DAB cubic term**		**DAB : gen**	
Variable	Chisq	Pr (>Chisq)	Chisq	Pr (>Chisq)	Chisq	Pr (>Chisq)	Chisq	Pr (>Chisq)	Chisq	Pr (>Chisq)
cpdh1	54.83	1.24e-12 ***	161.51	8.50e-36 ***	74.69	5.49e-18 ***	55.85	7.82e-14 ***	36.36	1.27e-08 ***
cpdh2	34.52	3.19e-08 ***	130.85	3.86e-29 ***	48.64	3.08e-12 ***	33.53	7.00e-09 ***	20.44	3.65e-05 ***
Ole	41.00	1.25e-09 ***	198.94	6.31e-44 ***	6.20	1.27e-02	0.49	4.82e-01	77.51	1.48e-17 ***
Urs	35.22	2.24e-08 ***	211.88	9.79e-47 ***	82.38	1.12e-19 ***	53.87	2.14e-13 ***	54.03	1.85e-12 ***
thu1	39.82	2.25e-09 ***	142.79	9.85e-32 ***	2.24	1.34e-01	12.07	5.12e-04 **	54.81	1.25e-12 ***
thu2	40.67	1.47e-09 ***	133.15	1.22e-29 ***	9.70	1.84e-03 *	1.58	2.09e-01	35.76	1.72e-08 ***
cdhu3	22.26	1.47e-05 ***	127.34	2.22e-28 ***	47.16	6.54e-12 ***	61.26	4.99e-15 ***	44.65	2.02e-10 ***
cdhu5	31.51	1.44e-07 ***	137.12	1.67e-30 ***	52.77	3.75e-13 ***	67.96	1.67e-16 ***	60.62	6.87e-14 ***
cdhu6	24.39	5.07e-06 ***	133.15	1.22e-29 ***	54.16	1.84e-13 ***	68.64	1.18e-16 ***	48.46	2.99e-11 ***
cou4	7.40	2.47e-02	92.29	9.12e-21 ***	4.57	3.25e-02	11.47	7.08e-04 **	25.14	3.48e-06 ***

Summary of results (Chisq statistic and pvalue) for lipids from nectarine cuticular waxes analyzed by GC-MS for the year 2012 and 2 genotypes (Summergrand and Zéphir). Tested effects by GLMM nested models (significance level of 0.01).

Tested effect	genotype		DAB		DAB quadratic term	
Variable	Chisq	Pr (>Chisq)	Chisq	Pr (>Chisq)	Chisq	Pr (>Chisq)
C16FA	0.125	0.724	8.99	0.011	5.02	0.025
C18FA	1.30E-03	0.971	2.86	0.238	0.309	0.578
C18_1FA	3.14	0.077	59.3	1.27E-13 ***	0.147	0.701
C18_2FA	0.0514	0.821	3.47	0.176	0.920	0.337
C20FA	3.59	0.058	2.89	0.236	4.96E-03	0.944
C22FA	0.429	0.513	12.1	2.40E-03 *	6.96	8.32E-03 *
C24FA	0.952	0.329	32.6	8.23E-08 ***	4.60	0.032
C26FA	0.989	0.320	18.9	7.66E-05 ***	2.99	0.084
C28FA	0.926	0.336	7.53	0.023	2.38	0.123
C19Alk	0.497	0.481	27.7	9.51E-07 ***	1.94	0.163
C20Alk	26.9	2.11E-07 ***	85.2	3.09E-19 ***	50.8	1.01E-12 ***
C21Alk	0.366	0.545	35.4	2.05E-08 ***	0.516	0.473
C23Alk	0.0107	0.918	111.2	7.03E-25 ***	28.3	1.03E-07 ***
C24Alk	6.04	0.014	67.5	2.14E-15 ***	5.66	0.017
C25Alk	1.56E-03	0.969	73.0	1.40E-16 ***	0.799	0.371
C27Alk	1.44	0.230	99.4	2.56E-22 ***	1.94	0.163
C29Alk	0.232	0.630	60.1	9.01E-14 ***	0.175	0.676
C31Alk	4.71	0.030	19.3	6.50E-05 ***	10.8	1.01E-03 *
C18OH	4.23E-05	0.995	8.36	0.015	7.28	6.96E-03 *
C20OH	0.133	2.69E-04 **	8.69	0.013	1.63	0.202
C22OH	0.487	0.485	1.33	0.513	0.923	0.337
C23OH	12.3	4.63E-04 **	36.1	1.44E-08 ***	0.125	0.724
C24OH	6.79	9.16E-03 *	31.2	1.71E-07 ***	2.76	0.097
C26OH	0.383	0.536	35.3	2.11E-08 ***	2.74	0.098
C28OH	0.0656	0.798	29.2	4.49E-07 ***	0.040	0.842
C30OH	1.06	0.302	48.8	2.54E-11 ***	3.77	0.052
C18Aldh	0.790	0.374	19.9	4.62E-05 ***	7.57	5.94E-03 *
C22Aldh	2.25	0.134	42.9	4.76E-10 ***	10.3	1.34E-03 *
C24Aldh	9.31E-03	0.923	65.7	5.42E-15 ***	4.63E-03	0.946
C30Aldh	0.449	0.503	26.9	1.39E-06 ***	4.33	0.038
Stigmasta-3.5-diene	25.2	5.29E-07 ***	91.8	1.14E-20 ***	51.1	8.96E-13 ***
beta-sitosterol	0.242	0.622	28.6	6.09E-07 ***	3.82	0.051
Alkanes	0.0685	0.793	94.6	2.78E-21 ***	8.09E-03	0.928
Fatty acids	0.177	0.674	5.99	0.050	3.90	0.048
Fatty alcohols	0.204	0.652	16.4	2.79E-04 **	0.179	0.673
Fatty aldehydes	0.730	0.393	13.1	1.44E-03 *	0.0231	0.879
Phytosterols	3.34	0.067	5.18	0.075	0.199	0.656

Tested effect	DAB cubic term		DAB : gen	
Variable	Chisq	Pr (>Chisq)	Chisq	Pr (>Chisq)
C16FA	4.13	0.042	0.0113	0.915
C18FA	0.156	0.693	0.273	0.601
C18_1FA	1.75	0.186	5.23	0.022
C18_2FA	1.24	0.266	0.368	0.544
C20FA	9.76E-03	0.921	2.67	0.102
C22FA	5.78	0.016	3.58E-04	0.985
C24FA	7.26	7.04E-03 *	1.21	0.272
C26FA	4.54	0.033	2.26	0.133
C28FA	3.10	0.078	1.57	0.210
C19Alk	3.72	0.054	6.78E-03	0.934
C20Alk	42.6	6.77E-11 ***	22.8	1.83E-06 ***
C21Alk	2.73E-03	0.958	0.734	0.392
C23Alk	40.4	2.04E-10 ***	2.34E-03	0.961
C24Alk	1.92	0.166	5.99	0.014
C25Alk	3.91	0.048	1.22	0.270
C27Alk	3.00E-03	0.956	3.24	0.072
C29Alk	0.293	0.588	0.131	0.718
C31Alk	8.92	2.82E-03 *	9.90	1.65E-03 *
C18OH	6.69	9.72E-03 *	0.0392	0.843
C20OH	1.00	0.316	10.8	1.00E-03 *
C22OH	1.04	0.308	0.374	0.541
C23OH	0.105	0.746	7.97	4.75E-03 *
C24OH	1.15	0.284	3.29	0.070
C26OH	1.03	0.310	2.53	0.112
C28OH	0.623	0.430	1.72	0.190
C30OH	7.24	7.12E-03 *	5.37	0.021
C18Aldh	5.67	0.017	0.443	0.506
C22Aldh	14.4	1.50E-04 **	5.51	0.019
C24Aldh	0.913	0.339	0.168	0.682
C30Aldh	6.58	0.010	2.46	0.117
Stigmasta-3.5-diene	41.9	9.59E-11 ***	21.3	4.03E-06 ***
beta-sitosterol	1.99	0.158	0.0920	0.762
Alkanes	2.16	0.141	0.512	0.474
Fatty acids	4.43	0.035	0.334	0.563
Fatty alcohols	0.704	0.401	1.80	0.180
Fatty aldehydes	0.274	0.601	1.82	0.177
Phytosterols	0.445	0.505	2.36	0.125

Statistical results S2.

Polynomial effect in GLMM analysis was used to determine the shape of the relationships between surface conductance or infection probability and compounds. In case quadratic and cubic terms were significant. non-linear correlations were estimated using the nlcor function in R (GitHub 'ProcessMiner'). It returned nonlinear correlation estimate. corresponding adjusted p-value and a plot visualizing the nonlinear relationships.

Test of the effect of the cubic and quadratic terms of the total wax on the surface conductance evolution of 2 genotypes (Summergrand and Zéphir), from 2012 data

**Complete model**		**Df**	**AIC**	**logLik**	**Chisq**	**Chi Df**	**Pr(>Chisq)**
conductance ~ TotalWax + TotalWax² + TotalWax³ + (1 | genotype)		6	870	-429			
**Tested models**	**Tested effect**						
conductance ~ TotalWax + TotalWax³ + (1 | genotype)	quadratic term	5	927	-458	58.44	1	2.099e-14 ***
conductance ~ TotalWax + TotalWax² + (1 | genotype)	cubic term	5	918	-453	48.85	1	2.766e-12 ***

Results of the non-linear correlation analysis via nlcor function

**x**	Total waxes (µg.cm^{-2})
**y**	Surface conductance (cm.h^{-1})
**Non-linear correlation**	0.66
**adjusted p.value**	0.0040

Plot of non-linear relationships estimated by nlcor function

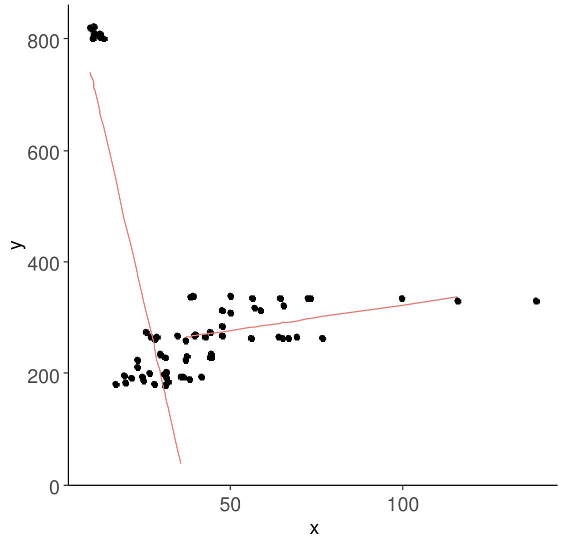

Test of the effect of the cubic and quadratic terms of sum of free terpenoids on the surface conductance evolution of the 3 genotypes (Summergrand, Zéphir and Magique), from 2012 and 2015 data

**Complete model**	**Tested effect**	**Df**	**AIC**	**logLik**	**Chisq**	**Chi Df**	**Pr(>Chisq)**
conductance ~ FreeTerp + FreeTerp² + FreeTerp³ + (1 | genotype)		6	1856	-922			
**Tested models**							
conductance ~ FreeTerp + FreeTerp³ + (1 | genotype)	quadratic term	5	1889	-940	35.08	1	3.168e-09 ***
conductance ~ FreeTerp + FreeTerp² + (1 | genotype)	cubic term	5	1901	-946	47.19	1	6.447e-12 ***

Results of the non-linear correlation analysis via nlcor function

**x**	Sum of free terpenoids (µg.cm^{-2})
**y**	Surface conductance (cm.h^{-1})
**Non-linear correlation**	0.32
**adjusted p.value**	8.09e-10

Plot of non-linear relationships estimated by nlcor function

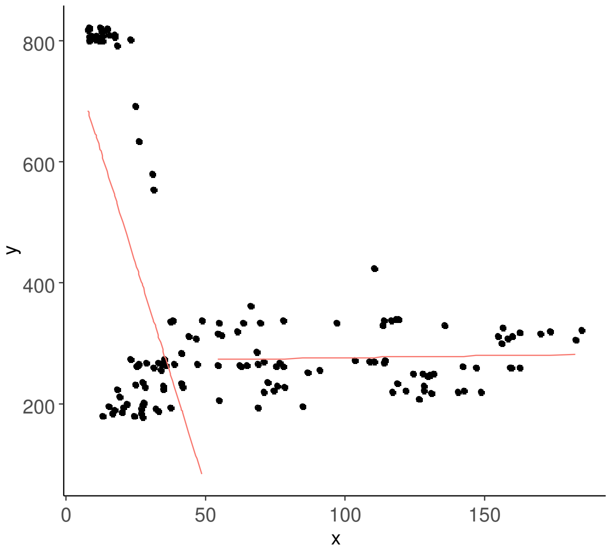

Test of the effect of the cubic and quadratic terms of sum of fatty aldehydes on the surface conductance evolution of 2 genotypes (Summergrand and Zéphir), from 2012 data

**Complete model**	**Tested effect**	**Df**	**AIC**	**logLik**	**Chisq**	**Chi Df**	**Pr(>Chisq)**
conductance ~ FattyAld + FattyAld² + FattyAld³ + (1 | genotype)		6	878	-433			
**Tested models**							
conductance ~ FattyAld + FattyAld³ + (1 | genotype)	quadratic term	5	887	-438	11.16	1	0.0008365 ***
conductance ~ FattyAld + FattyAld² + (1 | genotype)	cubic term	5	893	-442	17.29	1	3.207e-05 ***

Results of the non-linear correlation analysis via nlcor function

**x**	Sum of fatty aldehydes (µg.cm^{-2})
**y**	Surface conductance (cm.h^{-1})
**Non-linear correlation**	0.35
**adjusted p.value**	4.30e-06

Plot of non-linear relationships estimated by nlcor function

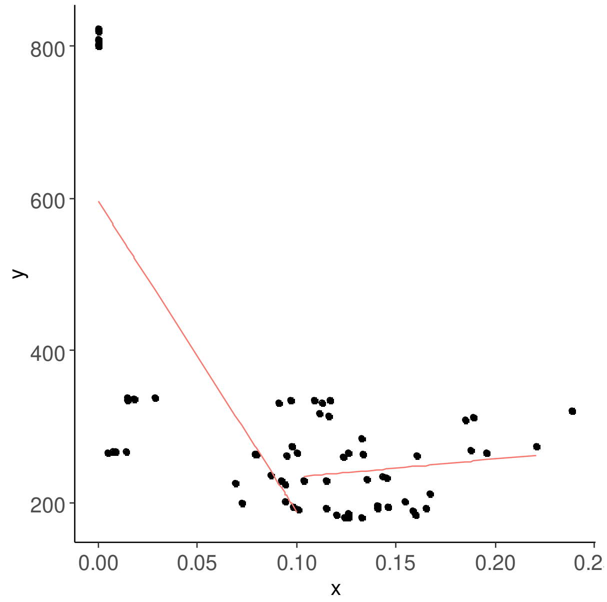

Test of the effect of the cubic and quadratic terms of sum of total waxes on the infection probability evolution of 2 genotypes (Summergrand and Zéphir), from 2012 data

**Complete model**	**Tested effect**	**Df**	**AIC**	**logLik**	**Chisq**	**Chi Df**	**Pr(>Chisq)**
infection ~ TotalWax + TotalWax²+ TotalWax³ + (1 | genotype)		6	95	-41.6			
**Tested models**							
infection ~ TotalWax + TotalWax³ + (1 | genotype)	quadratic term	5	109	-41.6	15.61	1	7.789e-05 ***
infection ~ TotalWax + TotalWax² + (1 | genotype)	cubic term	5	105	-47.7	12.18	1	0.000482 ***

Due to the low number of data. the nlcor function failed.

Test of the effect of the cubic and quadratic terms of sum of oleanolic and ursolic acids on the infection probability evolution of the 3 genotypes (Summergrand, Zéphir and Magique), from 2012 and 2015 data

**Complete model**	**Tested effect**	**Df**	**AIC**	**logLik**	**Chisq**	**Chi Df**	**Pr(>Chisq)**
infection ~ SumOlUrs + SumOlUrs²+ SumOlUrs³ + (1 | genotype)		6	206	-96.95			
**Tested models**							
infection ~ SumOlUrs + SumOlUrs³ + (1 | genotype)	quadratic term	5	215	-102.35	10.81	1	0.001009 **
infection ~ SumOlUrs + SumOlUrs² + (1 | genotype)	cubic term	5	212	-100.93	7.95	1	0.004794 **

Results of the non-linear correlation analysis via nlcor function

**x**	Sum of oleanolic and ursolic ($\mu g.cm^{-2}$)
**y**	Infection probability (%)
**Non-linear correlation**	0.35
**adjusted p.value**	0.015

Plot of non-linear relationships estimated by nlcor function

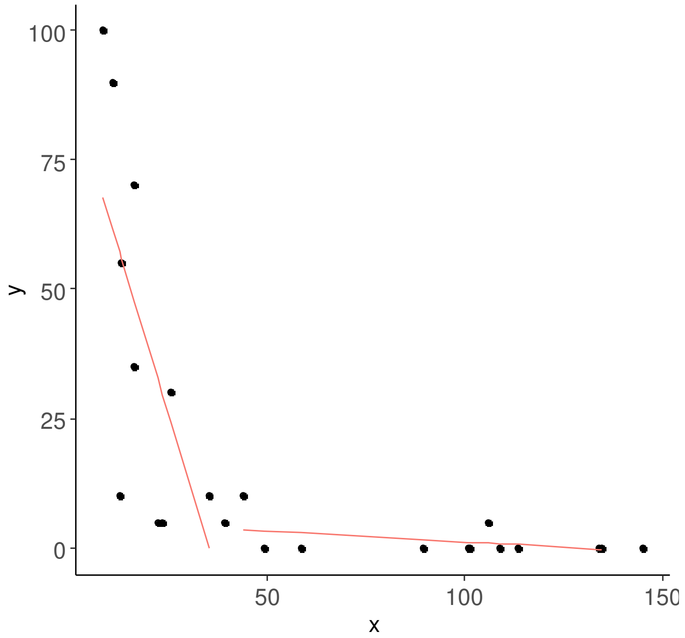

Supplement: eraa284_suppl_Supplementary_Material [file eraa284_suppl_supplementary_material.pdf]
